# Pluronic F-127 Enhances the Antifungal Activity of Fluconazole against Resistant *Candida* Strains

**DOI:** 10.1021/acsinfecdis.3c00536

**Published:** 2023-12-18

**Authors:** Katarzyna Malec, Aleksandra Mikołajczyk, Dominik Marciniak, Agnieszka Gawin-Mikołajewicz, Agnieszka Matera-Witkiewicz, Bożena Karolewicz, Urszula Nawrot, Yaroslav Z. Khimyak, Karol P. Nartowski

**Affiliations:** † Department of Drug Form Technology, Faculty of Pharmacy, Wroclaw Medical University, 211a Borowska Str, 50-556 Wroclaw, Poland; ‡ Screening Biological Activity Assays and Collection of Biological Material Laboratory, Wroclaw Medical University, 211a Borowska Str, 50-556 Wroclaw, Poland; § Department of Pharmaceutical Microbiology and Parasitology, Wroclaw Medical University, 211a Borowska Str, 50-556 Wroclaw, Poland; ∥ School of Pharmacy, University of East Anglia, Chancellors Drive, NR4 7TJ Norwich, U.K.

**Keywords:** resistant yeasts, Candida spp., efflux pump, Poloxamer, Pluronic, fluconazole

## Abstract

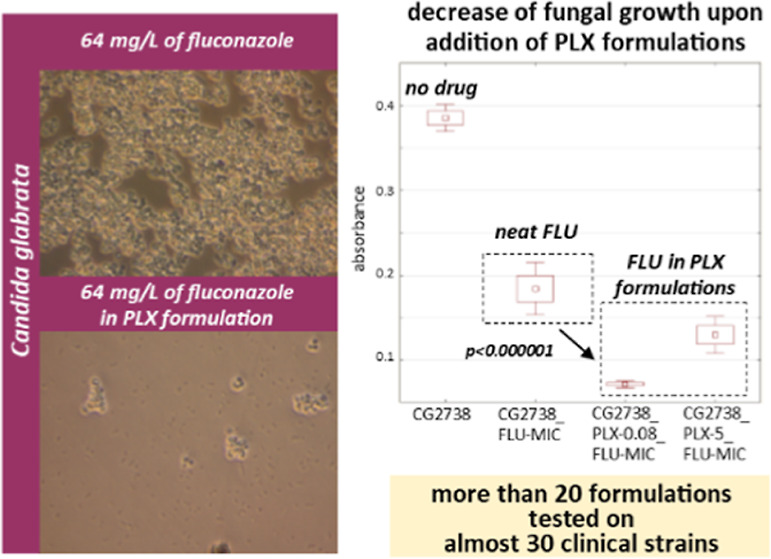

*Candida* strains as the most frequent causes of infections, along with their increased drug resistance, pose significant clinical and financial challenges to the healthcare system. Some polymeric excipients were reported to interfere with the multidrug resistance mechanism. Bearing in mind that there are a limited number of marketed products with fluconazole (FLU) for the topical route of administration, Pluronic F-127 (PLX)/FLU formulations were investigated in this work. The aims of this study were to investigate (i) whether PLX-based formulations can increase the susceptibility of resistant *Candida* strains to FLU, (ii) whether there is a correlation between block polymer concentration and the antifungal efficacy of the FLU-loaded PLX formulations, and (iii) what the potential mode of action of PLX assisting FLU is. The yeast growth inhibition upon incubation with PLX formulations loaded with FLU was statistically significant. The highest efficacy of the azole agent was observed in the presence of 5.0 and 10.0% w/v of PLX. The upregulation of the CDR1/CDR2 genes was detected in the investigated *Candida* strains, indicating that the efflux of the drug from the fungal cell was the main mechanism of the resistance.

## 1

**Figure 1:**
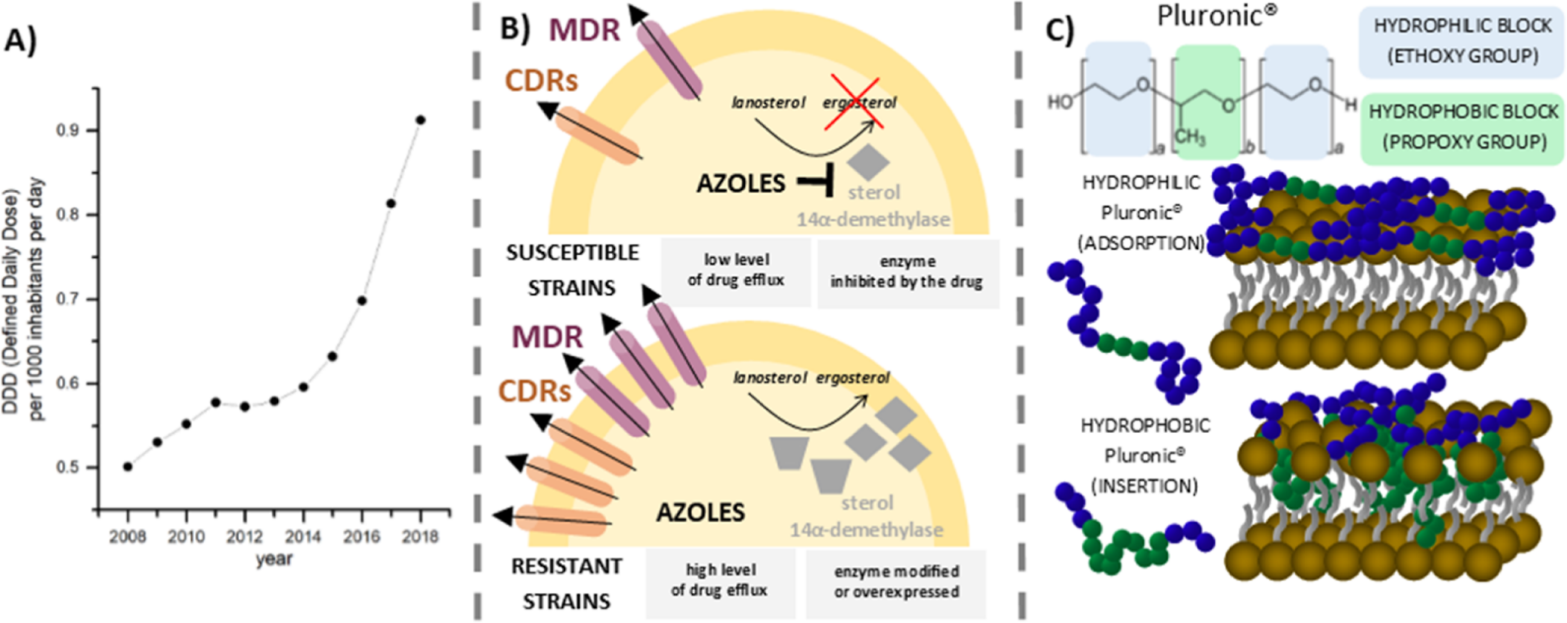
Consumption of antifungal drugs worldwide^17^ (A), Candida mechanisms of resistance to azoles^18^ (B), and mechanism of binding Pluronics to the cell membrane^16^ (C).

Fungal pathogens cause chronic conditions such as chronic mucocutaneous candidiasis, asthma, or chronic pulmonary aspergillosis, as well as life-threatening diseases such as fungaemia, pneumonia, or meningitis.^1^ Benedict et al. estimated that in 2017, the treatment of fungal diseases cost more than $7.2 billion, including a total cost of $1.4 billion for hospitalizations due to *Candida* infections (*n* = 26,735).^2^ A constant increase of systemic and topical fungal infections has been observed over the past decade, with an increasing number of recurrent infections such as oral and vaginal candidiasis.^1,3,4^ These can be caused by multidrug-resistant strains of fungi or non-*Candida albicansCandida* (NCAC) that are naturally resistant to commonly used treatments.^5^ As a consequence, total consumption of antifungal drugs has increased over the years worldwide (Figure 1A).^6,17^

In vulnerable groups of patients (e.g., treated with immunosuppressants), fungal infections are associated with high mortality. Therefore, early diagnosis and the selection of a safe and effective antifungal treatment determine the success of the therapy.^7^ As antifungal treatments are limited to only a few chemical classes, including azoles, echinocandins, polyenes, and flucytosine, increasing drug resistance is observed across clinically isolated fungal strains.^1^ The resistance phenomenon is particularly important for azole derivatives, which are the most popular antifungals used for prophylaxis as well as empirical and directed therapy. Due to the limited options for antifungal therapies, the emergence of multidrug-resistance strains can have devastating effects on patient outcomes. Therefore, the search for new antifungal agents or new antifungal formulations that can help overcome multidrug resistance in fungi is of paramount importance for the future development of antimicrobial treatments.

Representatives of the *Candida* genus have developed various mechanisms of resistance to azoles (Figure 1B). One of them is an active efflux of the drug from the fungal cell by membrane transporters. Two main classes of efflux pumps have been identified in *Candida* strains, namely, ABC proteins and MFS pumps. Overexpression of genes encoding membrane proteins (e.g., CDR1, CDR2, CDR3, CDR4, CDR11, MDR1, and FLU1) results in enhanced azoles efflux.^8^ Another resistance mechanism involves alterations in an enzyme that is a target for azoles, namely, sterol 14α-demethylase encoded by the ERG11 gene. This enzyme is involved in the synthesis of ergosterol, which is an essential component of the structure of fungal cell membranes. A point mutation in the gene leads to the expression of a protein that is characterized by a binding site of lower affinity.^9^ Therefore, evaluating the mechanism of resistance for clinical *Candida* isolates might result in the design of more effective pharmaceutical formulations against fungal infections.

Fluconazole is a representative of azoles, registered by the European Medicine Agency as tablets and capsules (dose 50–200 mg), the solution for infusion (2 mg/mL), syrup (5 mg/mL), and suspension at concentrations of 10 and 40 mg/mL.^10^ The marketed products are dedicated to systemic use only, with 0.5% Elazor gel registered in Italy being the only exception authorized for topical application in the European Union.^10^ Fluconazole is characterized by high molecular flexibility; therefore, it is prone to form various polymorphs^11^ that might affect its bioavailability in the formulation. For patients suffering from fungal keratitis, the solution for infusion can be compounded into eye drops as part of the course of personalized therapy.^12^ The topical route of administration could limit the systemic use of fluconazole and related side effects and the development of fluconazole-resistant strains. The possibilities include semisolid and liquid forms administered topically, such as gels, sprays, foams, or ointments that can be applied to the skin, mucous membranes, as well as into the eye. Such novel fluconazole formulations would primarily benefit patients vulnerable to recurrent fungal infections associated mainly with immunosuppressive or oncological treatments.^13,14^

It has been reported that some classes of polymers, in addition to their primary function as excipients, may exhibit additional properties affecting the final pharmacological effect of the applied formulation.^15^ Some polymers are characterized by intrinsic pharmacological activity, e.g., immunostimulating, antibacterial, antifungal, or antioxidant. Moreover, polymers themselves or conjugated with enzyme–inhibitor might inhibit certain enzymes, e.g., (chymo)trypsin, elastase, peptidase, and nuclease. They can interact with enzymes involved in drug metabolism (cytochrome P450 and CYP450), resulting in increased or decreased activity of some isoforms. They might also act as efflux pump inhibitors, resulting in a higher sensitivity of the cells to the treatment.^15^ With regards to Pluronics (PLX), inhibitory activity against ABC transporters such as Pgp (P-glycoprotein), MDR (multidrug resistance-associated protein), and BCRP (breast cancer resistance protein) has been reported.^16^ The proposed mechanism of binding to the cell membrane depends on the properties of the block copolymer (Figure 1C).^16^ The structure of the copolymer, i.e., the hydrophilic/hydrophobic balance, determines the mechanism of its binding with the cell surface. Hydrophilic Pluronics absorb mainly at the membrane surface, whereas hydrophobic Pluronics are more likely to penetrate into the lipid bilayer. The insertion is explained by the interaction of propoxy blocks with fatty acid residues and ethoxy groups with polar groups in the lipid bilayer. Alterations in the lipid microenvironment may facilitate membrane transport of small molecules (e.g., drugs).

The aim of the study was to investigate whether the addition of PLX can increase the susceptibility of resistant *Candida* strains to FLU and whether there is a correlation between block polymer concentration and the antifungal efficacy of FLU when assisted by PLX. Fluconazole was selected as the model drug due to the increasing resistance of *Candida* strains to this drug and a lack of FLU topical formulations on the market. We have selected amphiphilic Pluronic F-127 that has a PPO/PEO ratio equal to ca. 0.3 (respective block lengths of PEO–PPO–PEO are 100–65–100)^19^ to form micellar solutions and solutions gelling at various temperatures loaded with different FLU concentrations.

This work, for the first time, describes the enhanced activity of FLU in the presence of PLX against FLU-resistant *Candida* strains. To the best of our knowledge, this is the first extensive study covering a wide range of PLX concentrations, including the mechanistic considerations of the increased antimicrobial activity of the investigated formulations.

## 2 Results

### 2.1 PLX and FLU Formulations

We studied materials with increasing Pluronic F-127 concentrations, namely from slightly above the critical micellization concentration, CMC (0.08% w/v)^20^ to 25.0% w/v. This series comprises a variety of formulations differing from each other in terms of their rheological properties. The detailed explanation regarding the concentration range of all the components is presented in Supporting Information, Section S1. They included both micellar solutions and solutions gelling at various temperatures (Table 1). Such a wide range of polymer concentrations enabled us to establish whether the polymer concentration might be a factor affecting the potential antifungal mode of action of the polymer.

**Table 1:** Samples Investigated in the Study

formulation	Pluronic F-127 [% w/v, mM]	fluconazole [mM]	verapamil [mM]
PLX-0.08_FLU-S	0.08(0.063)	15.5	
PLX-0.1_FLU-S	0.1(0.079)	15.6	
PLX-0.15_FLU-S	0.15(0.12)	15.7	
PLX-0.5_FLU-S	0.5(0.40)	16.0	
PLX-1_FLU-S	1.0(0.79)	16.5	
PLX-5_FLU-S	5.0(4.0)	19.2	
FLU-MIC		0.0016–0.052^a^	
		0.013–0.42^b^	
PLX-0.08	0.08(0.063)		
PLX-0.08_FLU-MIC	0.08(0.063)	0.0016–0.052^a^	
		0.013–0.42^b^	
PLX-5	5.0(4.0)		
PLX-5_FLU-MIC	5.0(4.0)	0.0016–0.052^a^	
		0.013–0.42^b^	
PLX-10_FLU-MIC	10.0(7.9)	0.013–0.42^b^	
FLU-4		4.1	
FLU-8		8.2	
FLU-12		12.2	
FLU-15		14.7	
PLX-10	10.0(7.9)		
PLX-10_FLU-4	10.0(7.9)	4.1	
PLX-10_FLU-8	10.0(7.9)	8.2	
PLX-10_FLU-12	10.0(7.9)	12.2	
PLX-10_FLU-15	10.0(7.9)	14.7	
PLX-15	15.0(11.9)		
PLX-15_FLU-4	15.0(11.9)	4.1	
PLX-15_FLU-8	15.0(11.9)	8.2	
PLX-15_FLU-12	15.0(11.9)	12.2	
PLX-15_FLU-15	15.0(11.9)	14.7	
PLX-20	20.0(15.9)		
PLX-20_FLU-4	20.0(15.9)	4.1	
PLX-20_FLU-8	20.0(15.9)	8.2	
PLX-20_FLU-12	20.0(15.9)	12.2	
PLX-20_FLU-15	20.0(15.9)	14.7	
PLX-25	25.0(19.8)		
PLX-25_FLU-4	25.0(19.8)	4.1	
PLX-25_FLU-8	25.0(19.8)	8.2	
PLX-25_FLU-12	25.0(19.8)	12.2	
PLX-25_FLU-15	25.0(19.8)	14.7	
VER			0.10
VER_FLU-MIC		0.0016–0.052^a^	0.10
		0.013–0.42^b^	

a For susceptible *Candida* strains.

b For resistant *Candida* strains.

**Figure 2:**
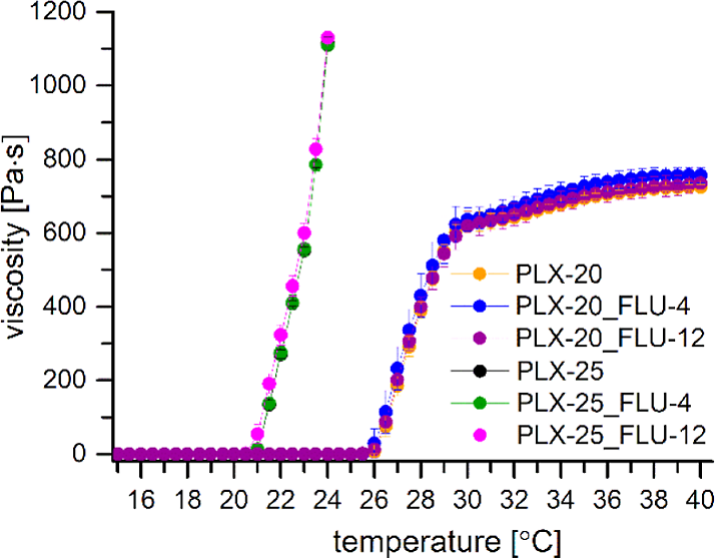
Effect of temperature on the viscosity of the investigated formulations: (a) PLX-20 and (b) PLX-25 with and without FLU addition; mean and standard errors are calculated from three experiments, shear rate was 0.192 s^–1^.

The viscosity–temperature plot of Pluronic F-127 formulations (20.0 and 25.0% w/v) showed sol–gel transition at 25.8 ± 0.2 and 21.3 ± 0.0 °C, respectively (Figure 2, Table 2). Although the sol–gel transition temperature decreased with increasing polymer concentration, it was not affected by the addition of fluconazole (Table 2). The phase transition temperatures were below the temperature of the human body (36.6 °C) and human body surface (34.0 °C); therefore, the formulations with 20.0 and 25.0% w/v polymer content might be effectively administered topically or into body cavities when developed into the final pharmaceutical product.

**Table 2:** Temperature of the Sol–Gel Phase Transition of the Investigated Formulations Based on PLX-20 and PLX-25^a^

formulation	*T*_gel_ [°C]
PLX-20	25.8 ± 0.2
PLX-20_FLU-4	25.8 ± 0.2
PLX-20_FLU-12	26.0 ± 0.2
PLX-25	21.3 ± 0.0
PLX-25_FLU-4	21.3 ± 0.1
PLX-25_FLU-12	21.1 ± 0.1

a Mean and standard errors are calculated from three repeat experiments, and the shear rate was 0.192 s^–1^.

At PLX concentrations of 10.0 and 15.0% w/v, the sol–gel transition was not observed (Supporting Information, Figure S1). PLX-20 compositions were liquid at room temperature. This might facilitate their application if formulated into the final drug form, such as eye drops or sprays with enhanced adhesive properties to the cornea, oral mucosa, or skin. The summary of the state of Pluronic F-127 formulations at particular polymer concentrations is presented in Supporting Information, Table S2.

### 2.2 Drug Content in Micellar Solutions of Pluronic F-127

As Pluronic F-127 increases the solubility of the drugs,^20−22^ we have studied whether micellar solutions up to 5.0% w/v loaded with fluconazole at its maximum solubility at a particular polymer concentration remained stable over prolonged storage at room temperature. Our results indicate that fluconazole was dissolved within a polymer solution throughout a whole range of investigated concentrations over 84 days (Supporting Information, Section S3). The drug content within the formulations remained within 100 ± 3.0%, depending on the sample.

### 2.3 Antifungal Activity of Pluronic F-127 Micellar Solutions of FLU

**Figure 3:**
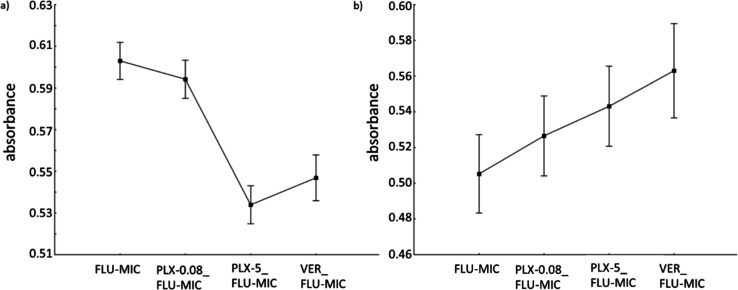
(a) Results of the broth microdilution method applied to resistant *Candida glabrata* strains derived from the multivariate analysis of variance (MANOVA). Current effect: *F*(3, 853) = 52.173, *p* < 0.00001, mean value ± 95% CI. (b) Results of the broth microdilution method applied to susceptible *Candida glabrata* strains derived from the MANOVA. Current effect: *F*(3, 756) = 4.0753, *p* = 0.00693, mean value ±95% CI.

FLU-MIC, PLX-0.08, PLX-0.08_FLU-MIC, PLX-5, and PLX-5_FLU-MIC formulations were used in microbiological studies using the broth microdilution method. The addition of micellar solutions of PLX loaded with FLU to resistant *Candida glabrata* resulted in a statistically significant decrease in the absorbance value for both investigated PLX concentrations, i.e., slightly above the CMC and at 5.0% w/v (Figure 3a and Table S4 in Supporting Information, FLU-MIC vs PLX-0.08_FLU-MIC, *p* = 0.000005; FLU-MIC vs PLX-5_FLU-MIC, *p* < 0.000001). Among the investigated strains, the largest decrease in absorbance value was observed for PLX-5_FLU-MIC formulations applied to *C. glabrata* strains 2586, 2738, 2853, 1973, 1467, and 2124 (Supporting Information, Figure S5 and Table S4, *p* = 0.000003, *p* < 0.000001, *p* < 0.000001, *p* < 0.000001, *p* = 0.000001, and *p* = 0.000001, respectively). The detailed statistical analysis for all the investigated *Candida* strains (*Candida krusei*, *C. albicans*, *C. glabrata*, and *Candida tropicalis*) is presented in the Supporting Information (Figure S6 and Table S5).

In contrast, susceptible strains of *Candida* did not show a statistically significant decrease in the absorbance value, indicating that PLX formulations are indeed interfering with the resistance mechanisms of *Candida* strains (Figure 3b and Table S6 in Supporting Information, FLU-MIC vs PLX-0.08_FLU-MIC, *p* = 0.320145, and FLU-MIC vs PLX-5_FLU-MIC, *p* = 0.060342, and Supporting Information, Figure S7 and Table S6).

MIC values in the five studied resistant *Candida* strains (*C. krusei* ATCC 6258, *C. albicans* 3057, *C. glabrata* 2586, *C. glabrata* 2738, and *C. glabrata* 2853) also decreased upon the addition of either PLX-0.08_FLU-MIC or PLX-5_FLU-MIC micellar solutions of FLU (Table 3). In the four *Candida* strains susceptible to increased exposure to fluconazole (namely *C. glabrata* 1640, 634, 635, and 2453), a decrease in MIC values was observed after treatment with the PLX-0.08_FLU-MIC formulation (Table 4).

**Table 3:** Antifungal Activity of Fluconazole, Fluconazole Loaded into Block Polymer Pluronic F-127 at 0.08 and 5.0% w/v, and Fluconazole in the Presence of Verapamil Hydrochloride in the *Candida* Strains Resistant to Fluconazole

	MIC_50_ [mg/L]			
strain	FLU-MIC	PLX-0.08_FLU-MIC	PLX-5_FLU-MIC	VER_FLU-MIC
*C. krusei* ATCC 6258	32	16^a^	16^a^	64
*C. albicans* ATCC MYA-574	>64	>64	>64	>64
*C. albicans* ATCC 64124	>64	>64	>64	>64
*C. albicans* 1444	>64	>64	>64	>64
*C. albicans* 3057	64	32^a^	32^a^	16^a^
*C. albicans* 3089	32	32	b	64
*C. tropicalis* 3151	>64	>64	b	8^a^
*C. glabrata* 2586	64	32^a^	32^a^	32^a^
*C. glabrata* 2738	64	64^a^	32^a^	32^a^
*C. glabrata* 140	64	64	64	32^a^
*C. glabrata* 769	64	64	64	32^a^
*C. glabrata* 773	64	64	64	64
*C. glabrata* 1941	64	64	64	64
*C. glabrata* 1973	64	64	64	64
*C. glabrata* 2342	64	64	64	64
*C. glabrata* 3154	64	64	64	64
*C. glabrata* 1467	64	64	64	64
*C. glabrata* 2853	32	16^a^	16^a^	16^a^
*C. glabrata* 3010	32	32	32	32
*C. glabrata* 137	32	32	32	32
*C. glabrata* 2124	32	32	32	64
*C. glabrata* 3081	32	32	32	32

a A decrease in MIC values in comparison to fluconazole solutions.

b 5.0% Pluronic F-127 led to ≥50% growth inhibition, thus being excluded from MIC evaluation.

**Table 4:** Antifungal Activity of Fluconazole, Fluconazole Loaded into Block Polymer Pluronic F-127 at 0.08 and 5.0% w/v, and Fluconazole in the Presence of Verapamil Hydrochloride in the *Candida* Strains Susceptible to the Increased Exposure to Fluconazole

	MIC_50_ [mg/L]			
strain	FLU-MIC	PLX-0.08_FLU-MIC	PLX-5_FLU-MIC	VER_FLU-MIC
*C. glabrata* 2665	16	16	16	16
*C. glabrata* 1004	8	8	8	16
*C. glabrata* 1640	4	2^a^	4	8
*C. glabrata* 634	4	2^a^	4	8
*C. glabrata* 635	4	2^a^	4	8
*C. glabrata* 393	4	4	4	8
*C. glabrata* 2453	2	1^a^	4	8
*C. glabrata* 2903	2	2	4	8

a A decrease in MIC values in comparison to fluconazole solutions.

Block copolymers alone without the addition of fluconazole, namely, PLX-0.08 and PLX-5, did not cause ≥50% growth inhibition (except for *C. albicans* 3089 and *C. tropicalis* 3151 at 5.0% w/v polymer; thus, these two strains were excluded from the MIC evaluation and statistical analysis for PLX-5_FLU-MIC formulations).

Furthermore, addition of verapamil (series VER_FLU-MIC) induced a statistically significant decrease in the growth of resistant *C. glabrata* (Figure 3a and Table S4 in Supporting Information, FLU-MIC vs VER_FLU-MIC, *p* < 0.000001). This may indicate that the FLU resistance is a result of ABC and/or MDR efflux pump overexpression. A statistically significant decrease in absorbance values after incubation of yeasts with verapamil-fluconazole formulations was observed for the following strains (see Supporting Information, Figure S5 for details): *C. glabrata* 2586 (*p* = 0.005267), 2738 (*p* = 0.002483), 2853 (*p* = 0.000025), 1973 (*p* = 0.001151), and 1467 (*p* = 0.003504). The detailed statistical analysis involving all the studied *Candida* strains (*C. krusei*, *C. albicans*, *C. glabrata*, and *C. tropicalis*) showed similarly a statistically significant decrease in absorbance value (with respect to the FLU-MIC series, see Figure S6 and Table S5). In contrast, FLU-susceptible *Candida* strains did not show a decrease in absorbance when treated with VER-containing samples (Figure 3b, FLU-MIC vs VER_FLU-MIC, *p* = 0.111595 and Supporting Information, Figure S7 and Table S6). In 7 strains resistant to fluconazole, MIC decreased upon addition of verapamil (series VER_FLU-MIC, Table 3). The largest decrease of the MIC value upon addition of verapamil was observed for *C. albicans* 3057, *C. tropicalis* 3151, and *C. glabrata* 2853. In the case of strains susceptible to increased exposure to fluconazole, the addition of verapamil did not trigger a decrease in MIC values (Table 4). Control experiments with verapamil did not inhibit yeast growth, regardless of the strain (VER sample).

Among the investigated resistant strains, *C. albicans* 3057, *C. glabrata* 2586, *C. glabrata* 2738, and *C. glabrata* 2853 were characterized by a decrease of the MIC value upon addition of both Pluronic and verapamil, which might suggest that two of them share a similar mechanism of action. Hence, we suppose that the polymer might interfere to some extent with the efflux pump, allowing enhanced accumulation of the drug inside cells.

### 2.4 Effect of Pluronic F-127 Concentration on Antifungal Activity of FLU

As some differences in the susceptibility of yeasts to fluconazole were observed between the series composed of 0.08 and 5.0% w/v Pluronic F-127, the antifungal effect of higher polymer concentrations was investigated further. Since the increased concentration of polymer results in increased viscosity of the Pluronic F-127 solution^23^ that could hinder the use of the broth microdilution method, the cup plate method was used for the investigation of the antifungal activity of PLX formulations at 10.0, 15.0, 20.0, and 25.0% w/v loaded with fluconazole at four different concentrations from 4 to 15 mM (PLX-10_FLU-4–15, PLX-15_FLU-4–15, PLX-20_FLU-4–15, and PLX-25_FLU-4–15, respectively) and fluconazole solutions without polymer addition (samples FLU-4–15).

Solutions of Pluronic F-127 loaded with fluconazole exhibited antifungal activity when tested by the cup plate method. This suggests that the addition of block polymer, even at higher concentrations did not hinder the antifungal activity of fluconazole, similarly to the results obtained by the broth microdilution method. Fifteen resistant strains were selected for cup plate method studies (*C. krusei* ATCC 6258, *C. albicans* 3057, and *C. glabrata* 2586, 2738, 2853, 3010, 1467, 2124, 137, 140, 1941, 1973, 2342, 3154, and 773). The diameter of the zone of growth inhibition is summarized in Table 5. It is important to note that Pluronic F-127 without the addition of fluconazole, regardless of the polymer concentration (series PLX-10, PLX-15, PLX-20, and PLX-25), applied to the wells did not produce the inhibition zone.

**Table 5:** Antifungal Activity of Fluconazole and Fluconazole Loaded into Pluronic F-127 at 10–20% w/v Concentrations Evaluated by the Cup Plate Method in Resistance to Fluconazole *Candida* Strains (Mean Values Presented with Standard Deviation)^a^

	inhibition zone [mm]							
strain	FLU-4	FLU-8	FLU-12	FLU-15	PLX-10_FLU-4	PLX-10_FLU-8	PLX-10_FLU-12	PLX-10_FLU-15
*C. k.* ATCC 6258	7.0 ± 0.0	14.8 ± 1.9	20.4 ± 1.8	22.8 ± 1.2	12.8 ± 0.4	22.4 ± 0.5	24.2 ± 0.8	26.2 ± 0.4
*C. a.* 3057	7.0 ± 0.0	21.3 ± 1.0	24.3 ± 1.1	26.2 ± 1.2	19.4 ± 0.5	24.0 ± 0.5	26.5 ± 0.8	27.6 ± 0.5
*C. g.* 2586	7.0 ± 0.0	7.0 ± 0.0	14.6 ± 0.6	17.2 ± 0.9	7.0 ± 0.0	7.0 ± 0.0	18.0 ± 0.0	18.3 ± 0.6
*C. g.* 2738	7.0 ± 0.0	7.0 ± 0.0	16.3 ± 0.8	17.7 ± 0.5	7.0 ± 0.0	7.0 ± 0.0	18.2 ± 0.9	19.6 ± 1.2
*C. g.* 2853	7.0 ± 0.0	16.5 ± 1.1	19.2 ± 1.1	20.7 ± 0.4	7.0 ± 0.0	19.6 ± 0.5	22.5 ± 0.5	26.4 ± 0.5
*C. g.* 3010	7.0 ± 0.0	7.0 ± 0.0	15.3 ± 0.5	18.2 ± 0.7	19.1 ± 1.2	24.2 ± 1.2	27.6 ± 0.5	29.8 ± 0.5
*C. g.* 1467	7.0 ± 0.0	7.0 ± 0.0	7.0 ± 0.0	14.7 ± 0.6	7.0 ± 0.0	7.0 ± 0.0	18.0 ± 0.0	18.7 ± 0.4
*C. g.* 2124	7.0 ± 0.0	7.0 ± 0.0	16.7 ± 1.4	20.8 ± 1.0	7.0 ± 0.0	17.7 ± 0.4	20.3 ± 0.7	21.4 ± 0.9
*C. g.* 137	7.0 ± 0.0	7.0 ± 0.0	16.3 ± 1.3	18.2 ± 0.6	7.0 ± 0.0	7.0 ± 0.0	18.6 ± 1.3	20.1 ± 1.3
*C. g.* 140	7.0 ± 0.0	7.0 ± 0.0	17.6 ± 0.5	19.9 ± 0.5	7.0 ± 0.0	21.5 ± 0.0	20.3 ± 1.4	20.5 ± 0.6

strain	PLX-15_FLU-4	PLX-15_FLU-8	PLX-15_FLU-12	PLX-15_FLU-15	PLX-20_FLU-4	PLX-20_FLU-8	PLX-20_FLU-12	PLX-20_FLU-15
*C. k.* ATCC 6258	7.0 ± 0.0	17.2 ± 1.8	21.3 ± 0.7	22.9 ± 1.8	10.0 ± 0.9	16.6 ± 1.0	20.6 ± 1.3	21.2 ± 1.5
*C. a.* 3057	19.8 ± 0.4	23.1 ± 0.8	25.2 ± 0.4	26.8 ± 0.8	20.3 ± 0.5	24.1 ± 2.0	25.6 ± 2.4	25.9 ± 2.0
*C. g.* 2586	7.0 ± 0.0	7.0 ± 0.0	16.2 ± 0.8	17.3 ± 0.3	7.0 ± 0.0	7.0 ± 0.0	16.7 ± 0.3	18.0 ± 0.0
*C. g.* 2738	7.0 ± 0.0	14.3 ± 0.6	19.8 ± 0.8	17.7 ± 0.9	7.0 ± 0.0	7.0 ± 0.0	18.7 ± 0.8	17.2 ± 0.7
*C. g.* 2853	7.0 ± 0.0	19.3 ± 0.6	20.8 ± 0.3	20.9 ± 0.7	7.0 ± 0.0	16.7 ± 0.6	20.2 ± 0.6	20.2 ± 0.6
*C. g.* 3010	18.0 ± 0.5	24.1 ± 0.8	27.6 ± 0.5	29.3 ± 0.8	16.5 ± 0.5	21.9 ± 0.5	26.4 ± 1.0	26.3 ± 0.8
*C. g.* 1467	7.0 ± 0.0	7.0 ± 0.0	7.0 ± 0.0	17.3 ± 1.3	7.0 ± 0.0	7.0 ± 0.0	7.0 ± 0.0	15.7 ± 0.6
*C. g.* 2124	7.0 ± 0.0	7.0 ± 0.0	18.9 ± 1.0	18.8 ± 0.4	7.0 ± 0.0	7.0 ± 0.0	19.0 ± 1.4	19.4 ± 1.1
*C. g.* 137	7.0 ± 0.0	7.0 ± 0.0	17.7 ± 0.7	18.7 ± 0.8	7.0 ± 0.0	7.0 ± 0.0	18.6 ± 0.9	19.2 ± 0.5
*C. g.* 140	7.0 ± 0.0	7.0 ± 0.0	19.1 ± 0.6	20.4 ± 0.5	7.0 ± 0.0	7.0 ± 0.0	19.0 ± 0.6	19.7 ± 0.9

a *C. k.*—*C. krusei*; *C. a.*—*C. albicans*; *C. g.*—*C. glabrata*; if no inhibition zone was observed, the diameter of the well is indicated in the table.

For ten out of the 15 investigated strains, the inhibition zone increased when fluconazole was accompanied by block polymer regardless of the polymer concentration (samples PLX-10_FLU-4–15, PLX-15_FLU-4–15, and PLX-20_FLU-4–15) with a few exceptions (series PLX-20_FLU-15 incubated with *C. krusei* ATCC 6258, *C. glabrata* 2738, *C. glabrata* 2853, and *C. glabrata* 140).

**Figure 4:**
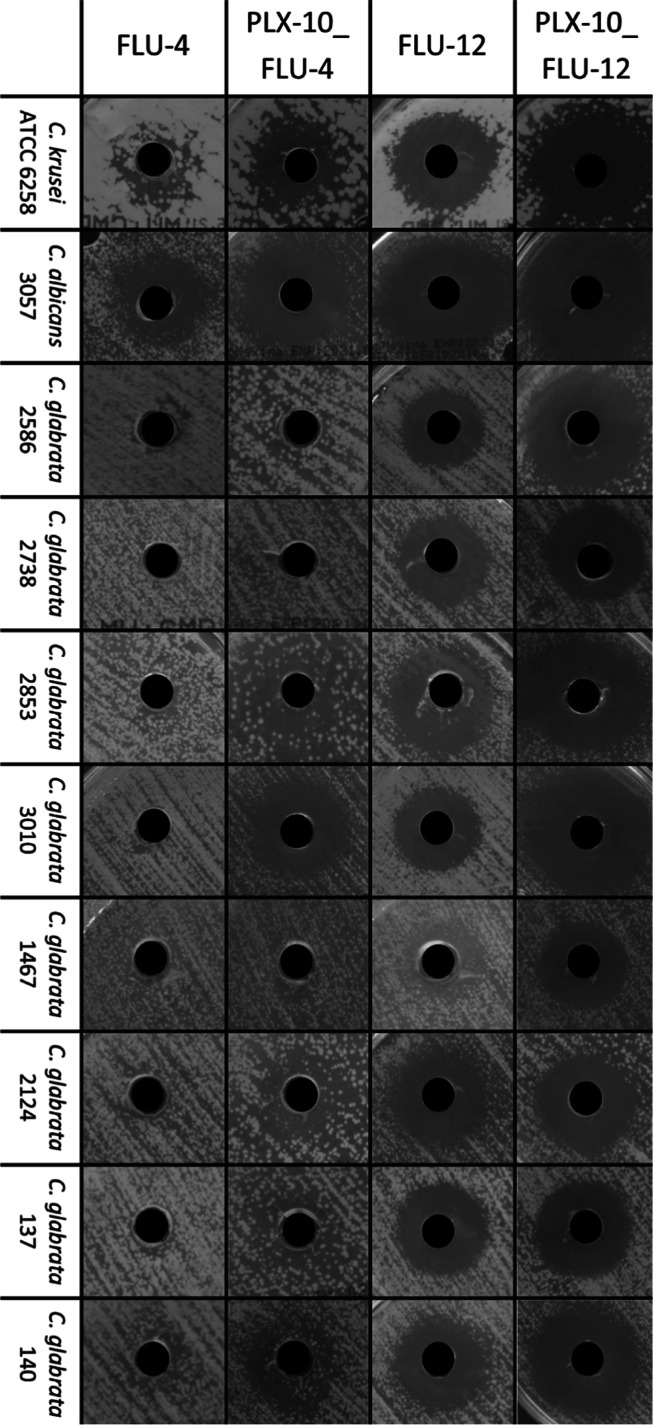
Images of inhibition zones of series PLX-10_FLU-4, PLX-10_FLU-12, FLU-4, and FLU-12 studied by the cup plate method.

The highest increase in the diameter of the zone of inhibition, as compared to the fluconazole solutions, was observed for PLX-10_FLU-4–15 formulations (Figure 4). In the series PLX-25_FLU-4–15 with 25.0% w/v Pluronic F-127 regardless of the strain, we observed a reduced inhibition zone to values lower than exhibited in the FLU-4–15 series (Supporting Information, Table S7). It is known that Pluronic F-127 at concentrations above 17% w/v undergoes a sol–gel transition at temperatures below 30 °C, resulting in the formation of viscous gels that may limit the drug diffusion into the agar. As presented in Table 2, 25.0% w/v Pluronic F-127 was gelling already at ca. 21 °C, indicating that even before the incubation with fungi, its penetration into agar was limited. Therefore, 25.0% w/v Pluronic F-127 formulations were excluded from the statistical analysis. The phase transition temperature might also be the explanation for the decreased inhibition zones in PLX-20_FLU-15 in the above-mentioned strains. In 5 investigated strains (*C. glabrata* 1941, 1973, 2342, 3154, and 773), no inhibition zone around wells at the plates was observed both for formulations with neat fluconazole and fluconazole with PLX.

**Figure 5:**
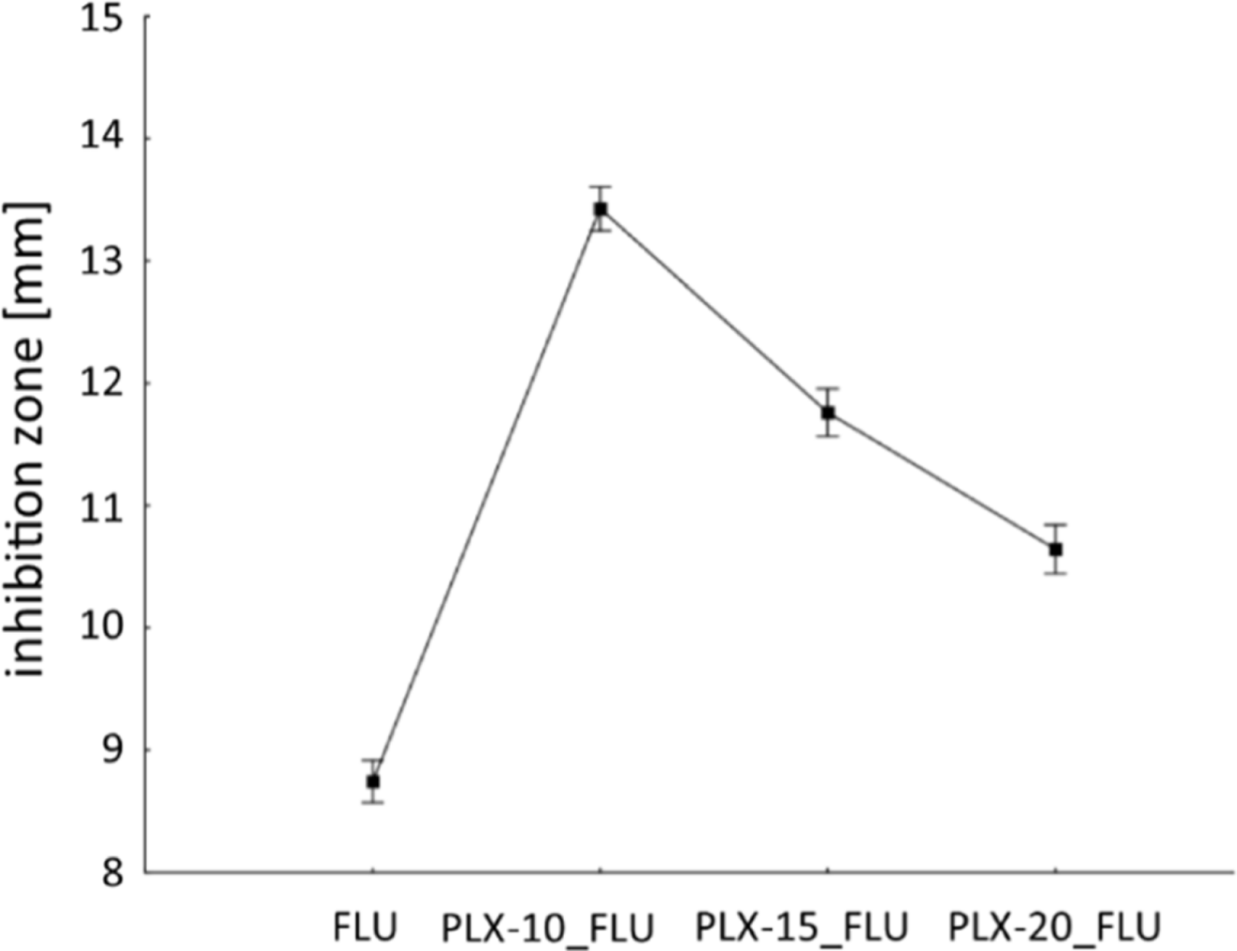
Results of the cup plate method applied to *Candida glabrata* strains derived from the MANOVA. Current effect: *F*(3, 786) = 474.48, *p* < 0.00001, mean value ± 95% CI.

For all *C. glabrata* strains, the inhibition zone was significantly larger when fluconazole was accompanied by block copolymers, regardless the copolymer concentration (Figure 5 and Table S8 in Supporting Information, FLU vs PLX-10_FLU, *p* < 0.000001, FLU vs PLX-15_FLU, *p* < 0.000001, and FLU vs PLX-20_FLU, *p* < 0.000001). The same phenomenon was observed when individual fluconazole concentrations loaded into polymer samples were considered (Supporting Information, Figure S8a and Table S8). The greatest changes were observed in the PLX-10_FLU series (Supporting Information, Figure S8b and Table S8). The statistical analysis of the combined data retrieved from experiments performed on all three different investigated resistant *Candida* strains showed a very similar pattern (*C. krusei*, *C. albicans*, and *C. glabrata*) and is presented in the Supporting Information (Figure S9 and Table S9).

### 2.5 Kinetics of the Antifungal Activity

As shown in Figure S10 in Supporting Information, the progressive increase of absorbance in wells with treated yeasts was observed over the course of time. The plateau was apparent, especially in the investigated *C. glabrata* strains after ca. 16–20 h of incubation. With increasing concentrations of fluconazole (in the range of 8–64 mg/L), a reduction of growth was observed; thus, growth curves of *Candida* showed dose-dependent inhibitory characteristics. When different concentrations of Pluronic F-127 were considered, 5% w/v caused faster inhibition of the yeast growth, as seen by the larger angle of the slope of the growth curves. This long-term kinetic study showed continuous inhibitory effects on fungal cells of the examined formulations, mostly starting at 5–6 h of incubation and lasting until 16–20 h of the experiment. It might be an indication of its prolonged action at the application site when developed into the final drug form.

### 2.6 ERG11, CDR1, and CDR2 Gene Expression Analysis

In order to determine the mechanism responsible for the enhanced resistance to fluconazole among the investigated *Candida* strains, the level of expression of the resistance-associated genes encoding drug transporters and sterol 14α-demethylase was established. The data on gene expression could provide an indication of the likely mode of action of Pluronic F-127. A detailed summary of the results from gene expression analysis in 15 *C. glabrata* strains resistant to fluconazole is given in the Supporting Information (Tables S10–S12).

Based on the real-time PCR results, the upregulation of the CDR1 and CDR2 genes is the major mechanism of resistance in the investigated strains (Table 6). Six out of 15 studied strains displayed a higher level of CDR1 expression in comparison to a reference isolate (2^–ΔΔ*CT*^ value in the range 1.19–1.95). Moreover, the other six analyzed strains demonstrated a significant increase of CDR1 transcript (2^–ΔΔ*CT*^ value between 2.01 and 8.85). Similarly, the CDR2 gene was overexpressed in nine strains as compared with the reference strain (2^–ΔΔ*CT*^ value in the range 1.03–1.75), and in five strains, a significant overexpression was detected (2^–ΔΔ*CT*^ value between 2.43 and 7.01). Each of the investigated strains displayed a higher expression level of either the CDR1 or CDR2 genes. Overall, the expression level of CDR1 genes was slightly higher compared to CDR2 genes.

**Table 6:** Summary of the Gene Expression Analysis among Resistant Strains Evaluated by Livak’s Method^24^ (Ranked by Increasing Level Expression of CDR1 and CDR2 Genes) in Regards to the Results Retrieved from the Broth Microdilution Method and the Cup Plate Method^a^

strain	ERG11	CDR1	CDR2	decrease of absorbance	increase of inhibition zone
*C. glabrata* 1941	+	+++	+++	√	×
*C. glabrata* 2738	–	+++	+++	√^b^	√
*C. glabrata* 1467	–	+++	+++	√	√
*C. glabrata* 1973	+	+++	+	√	×
*C. glabrata* 3010	–	+++	+	√	√
*C. glabrata* 2342	–	+++	+	√	×
*C. glabrata* 3154	–	+	+++	√	×
*C. glabrata* 3081	–	+	+++	×	n/a
*C. glabrata* 2586	–	+	+	√^b^	√
*C. glabrata* 137	–	+	+	√	√
*C. glabrata* 773	–	+	+	√	×
*C. glabrata* 769	–	0	+	×	n/a
*C. glabrata* 140	+	+	–	√	√
*C. glabrata* 2124	0	–	+	√	√
*C. glabrata* 2853	–	–	+	√^b^	√
*C. krusei* ATCC 6258		n/a		√^b^	√
*C. albicans* ATCC MYA-574		n/a		×	n/a
*C. albicans* ATCC 64124		n/a		×	n/a
*C. albicans* 1444		n/a		×	n/a
*C. albicans* 3057		n/a		√^b^	√
*C. albicans* 3089		n/a		√	n/a^c^
*C. tropicalis* 3151		n/a		√	n/a^c^

a Designation: (−) 2^–ΔΔ*CT*^ < 1; (0) 2^–ΔΔ*CT*^ = 1; (+) 1 < 2^–ΔΔ*CT*^ < 2; (+++) 2^–ΔΔ*CT*^ > 2; n/a—not applicable.

b Beside the absorbance decrease, MIC value decrease was also observed.

c Neat block polymer at 5.0% w/v inhibited yeast growth, thus being excluded from the cup plate method.

In the case of the ERG11 gene, only three out of 15 strains expressed the ERG11 gene at a higher level than a control strain, *C. glabrata* 1004 (the fold gene expression, 2^–ΔΔ*CT*^ in the range 1.25–1.31). The remaining strains exhibited a low level of ERG11 transcript (2^–ΔΔ*CT*^ value between 0.19 and 0.98), indicating ERG11 overexpression did not contribute to fluconazole resistance in the investigated strains to a large extent. Overall, *C. glabrata* 1941, 1467, and 2738 were characterized by the largest contribution of the pump efflux overexpression in resistance mechanism (2^–ΔΔ*CT*^ > 2 in case of both CDR1 and CDR2), followed by *C. glabrata* 1973, 2342, 3010, 3154, and 3081 (characterized by either a significant overexpression or higher expression level of studied CDR genes).

### 2.7 Microscopic Imaging

Selected samples were analyzed using microscopy in phase contrast to detect any changes in growth and monitor yeast morphology (Supporting Information, Figures S11 and S12). The decreased amount of yeast cells without any changes in their morphology was noticed in wells characterized by decreased absorbance after incubation of fungi with fluconazole and polymer.

**Figure 6:**
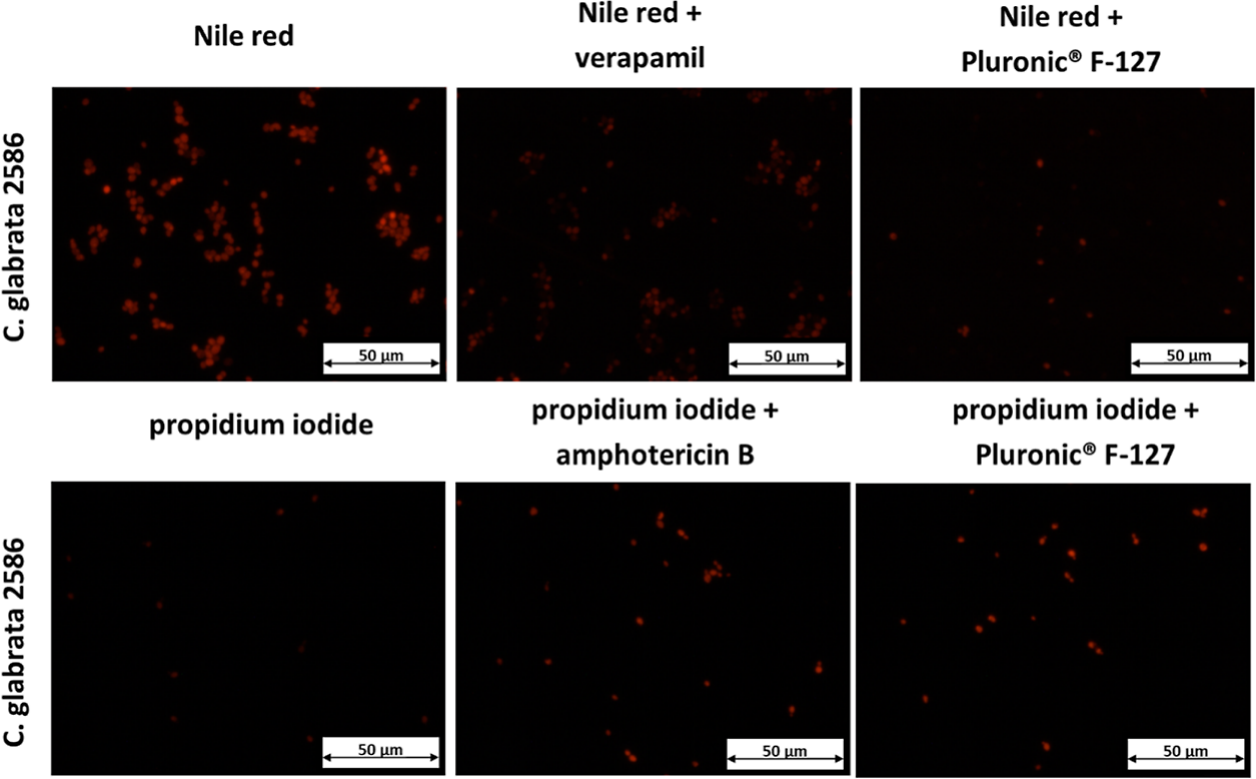
*C. glabrata* 2586 stained with Nile red and propidium iodide in the absence and presence of reference substances (verapamil and amphotericin B, accordingly) and Pluronic F-127.

The dye Nile red that fluoresces in a hydrophobic environment was chosen to track the efflux in *Candida* cells treated with Pluronic F-127 and a known efflux inhibitor, verapamil. Nile red is a substrate for many types of transporters, both ABC and MFS (Cdr1, Cdr2, and Mdr1, respectively).^25^ As shown in the images (Figure 6 and Supporting Information, Figure S13), *Candida* cells stained with Nile red, in the presence of both polymer and verapamil, exhibited fluorescence. However, the amount of stained cells in nontreated samples^26^ was larger in comparison to the treated cells, probably due to the inhibition of efflux pumps by both substances. It meant that the accumulation of the dye within cells was hindered by both verapamil and Pluronic F-127 affecting efflux pumps.

To evaluate whether polymers might interfere with the integrity of the fungal membranes as another possible way of acting, the cells incubated either with Pluronic F-127 or with amphotericin B were stained with propidium iodide. Propidium iodide is known to be a fluorescence dye that crosses damaged cell membranes and stains intracellular nucleic acids.^27^ Amphotericin B is a reference substance that, upon binding with cellular membranes, trigger the formation of pores, resulting in their death.^28^ Similarly to the case of Nile red staining, *Candida* cells, regardless of the substance used for incubation, exhibited fluorescence (Figure 6, and Supporting Information, Figure S14). The amount of stained cells treated with Pluronic F-127 was strain-dependent. The highest proportion was for the *C. glabrata* 2738 strain, exhibiting a significant overexpression of CDR1 and CDR2 genes, whereas the lowest was for *C. glabrata* 2853, characterized by a moderate overexpression of efflux pumps. As expected, Pluronic F-127 was less effective than a potent antifungal antibiotic when compared to amphotericin B-treated subculture. However, the results might indicate a linkage between the impaired function of the efflux pump and fungal membrane disruption when the mechanism of the action of the polymer considered.

## 3 Discussion

Taking into account the developing antifungal resistance among *Candida* strains, the application of excipients that exhibit antifungal potential, i.e., antimicrobial polymers as functional pharmaceutical formulation additives, is perceived as a favorable solution in future antifungal strategies. Only a few reports are concerned with the study on the potential antifungal activity of neat excipients or their synergism with the antifungal substance. A direct comparison of these data poses difficulties as these macromolecules have different molecular weights and methods of preparation. In addition, different pathogenic strains are investigated. Some of the few examples reporting potential antifungal activity toward *Candida* species include hyaluronic acid and chitosan. However, the mechanisms underlying such an action remain unclear. Data on excipients with potential antifungal activity against *Candida* strains or their synergistic effects with an antifungal drug are summarized in Table 7.

**Table 7:** Excipients with Potential Antifungal Activity against *Candida* Strains or with Synergistic Effects with Antifungal Drugs

type of polymer/active pharmaceutical ingredients	*Candida* strains	obtained effect	refs
Chitosan			
low molecular weight chitosan (LMWC)	105 clinical *Candida* isolates (*C. krusei*, *C. albicans*, *C. tropicalis*, *C. glabrata*), *C. krusei* ATCC 62258, *C. albicans* ATCC 64548 and ATCC 64550, *C. tropicalis* Rex MY1012, *C. glabrata* ATCC 90030, *S. cerevisiae* ATCC 9763, *C. lusitaniae* Rex CL2819, and *C. parapsilosis* ATCC 22019	MIC values of fluconazole assisted by chitosan were established, LMWC exhibited a significant antifungal activity, inhibiting over 89.9% of the clinical isolates examined (68.6% of which was completely inhibited), and the antifungal activity of LMWC increased at acidic pH, which might be due to the protonation of the amino group of glucosamine units of chitosan at pH 4.0, as the p*K*_a_ of LMWC is 6.3	(33)
high (HMW) and low molecular weight (LMW) chitosan	15 clinical isolates (*C. albicans*, *C. tropicalis* and *C. parapsilosis*), *C. albicans* ATCC 10231, *C. parapsilosis* ATCC 22019	fungal growth decreased with increasing molecular weight of chitosan for *C. tropicalis* and *C. parapsilosis*, while chitosan molecular weight did not modulate the effect against *C. albicans*	(34)
six commercial chitosans with distinct molecular weights and degrees of deacetylation	*C. albicans* SC5314, *C. tropicalis* MYA3404, and drug-resistant strains *C. albicans* and *C. tropicalis*	MIC values of chitosan and fluconazole along with fractional inhibitory concentration (FIC_index_) and inhibition zones showed great synergistic antifungal activity against the investigated *Candida* species	(29)
Hyaluronic Acid			
high molecular weight hyaluronic acid (1.8 MDa)	*C. albicans* ATCC 90028 and 90,029, *C. glabrata* ATCC 90030, and*C. parapsilosis* ATCC 22019	antifungal properties against *C. glabrata* and *C. parapsilosis*, with fungistatic activity reported to be dose-dependent	(35)
low molecular weight hyaluronic acid (1630 kDa)	*C. albicans* ATCC 10231, 18804, and 11,006	dose-dependent fungistatic activity against *C. albicans*	(36)

In general, antifungal polymers are characterized by cationic and hydrophobic regions that are expected to interact with anionic components of the cell wall, as well as microbial phospholipids and membranes. In studies performed by Lo et al., six various chitosans with specific molecular weights were applied in combination with fluconazole, showing a great synergistic fungicidal effect against susceptible and drug-resistant *C. albicans* and *C. tropicalis* both in liquid and agar media.^29^ Combination treatment exhibited a synergistic antifungal effect in both investigated drug-resistant strains (FIC_index_ < 0.5).^29^ Grimling et al. have demonstrated that compositions comprising clotrimazole and high-molecular-weight chitosan could be an effective solution in a topical antifungal formulation against non-*Candida albicansCandida* strains.^30^ The synergistic effect of clotrimazole and chitosan combinations was observed in tests carried out at pH 4 on *C. glabrata* strains.^30^ The inhibition of *C. glabrata* growth reached at least 90%, regardless of the drug/excipient weight ratio.^30^ Studies of the mechanism of action of both neat excipients and the developed formulations are rare. Shih et al. exceptionally attempted to evaluate the mechanism of the enhanced activity of chitosan, which is probably targeting the cell surface.^31^ The decrease in the expression of Ada2 genes was observed after exposure to 0.2% chitosan for 20 min and for 1 h in the wild-type strain *C. albicans* compared to polymer-untreated cells.^31^ Those genes are directly involved in the defining of the cell surface composition and its integrity by, e.g., regulating the MDR1 and CDR1 efflux pumps;^31^ therefore, the authors concluded that chitosan might have altered the integrity of the cell surface of *C. albicans*.

Since Pluronics were reported as one of the amphiphilic polymers that affect drug efflux transporters in cancer cells, resulting in sensitization and prevention of multidrug resistance,^16^ we have decided to investigate the effect of Pluronic F-127 on the *Candida* cells, which also express drug efflux pumps responsible for their resistance to fluconazole. It has been reported in the literature that Pluronic P85 (PEO–PPO–PEO with block lengths of 25–40–25)^19^ affected MDR cells already at concentrations below CMC (0.03 wt%).^32^ It was suggested that unimers are able to incorporate and translocate across the cellular membranes, where hydrophobic PPO chains of Pluronic are embedding into the membrane hydrophobic areas, causing so-called “membrane fluidization”, resulting in alterations of the membrane structure and decreasing its microviscosity.^32^ It has been proposed that the formation of micelles at higher concentrations of block copolymer might result in hiding these hydrophobic PPO chains in the micellar core, resulting in their diminished availability to affect the cellular membranes.^32^ However, as described in our recent work,^20^ molecular dynamics simulations along with experimental data showed an amphipathic microsegregated surface in Pluronic F-127 micelles with hydrophobic domains exposed to solvent instead of the typically accepted hydrophilic surface and hydrophobic core. This might be the reason for the fact that although the lowest studied concentration of Pluronic F-127 in the presented study was slightly above CMC, i.e., PLX-0.08_FLU-MIC, it preserved its efficacy in enhancing fluconazole activity, as described in the section “Antifungal 2.3”. It indicated that the formation of PLX micelles did not affect neither the antifungal activity of fluconazole nor the investigated properties of polymers. Moreover, the drug–micelle interactions at 5.0% w/v^35^ did not affect the activity of the drug.

In the presented study, individual strains were vulnerable to even small amounts of Pluronic (slightly above CMC) loaded with fluconazole, manifesting itself in a decrease of MIC value in the broth microdilution method. A more pronounced effect of statistical significance was observed in the case of a higher Pluronic concentration (5.0% w/v) with incorporated fluconazole. In strains investigated by the cup plate method, the lowest investigated concentration of block polymer (10.0% w/v) loaded with fluconazole led to an increased inhibition zone of yeast growth. In strains in which the inhibition zone did not appear, we assume that the applied concentrations of both fluconazole and polymer were not sufficient to attenuate the enhanced fungal resistance of those strains. Overall, the highest investigated polymer concentration (15–25% w/v) did not cause the largest inhibition of fungi growth when all the results derived from the cup plate method were considered. This could be explained by the increased viscosity under experimental conditions.

**Figure 7:**
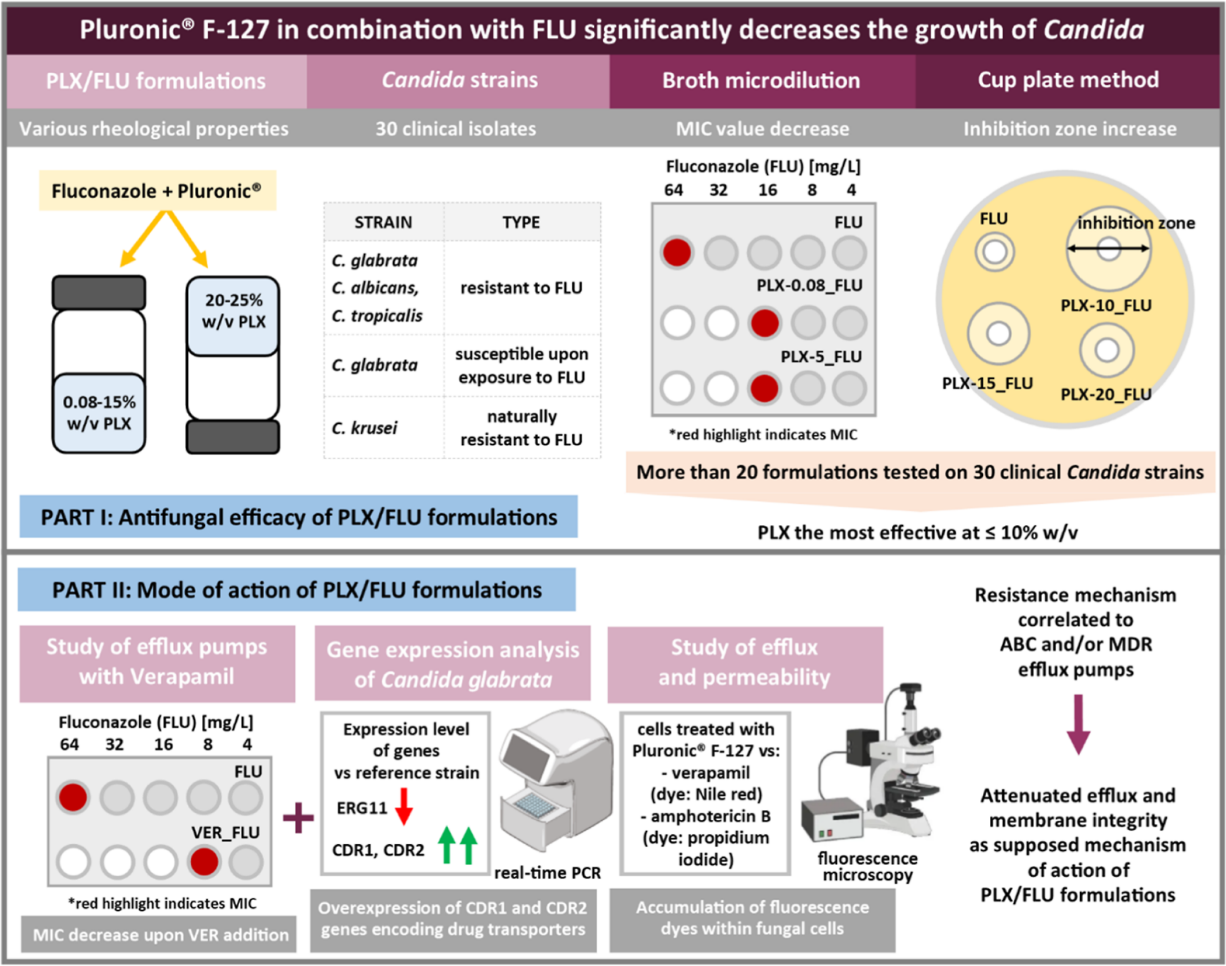
Enhanced antifungal efficacy of Pluronic F-127-based formulations loaded with fluconazole is correlated with their potential mode of action affecting membrane integrity and the function of membrane transporters.

As the addition of fluconazole to Pluronic F-127 led to the attenuated growth of the investigated yeasts when compared to neat fluconazole, the hypothesis for the reason for the observed enhanced fluconazole activity has been evaluated in several ways. The subsequent steps of the study are summarized in Figure 7. First, we have investigated whether fluconazole assisted by verapamil, a known example of the efflux pump inhibitor, would result in decreased yeast growth. A decrease in MIC value was observed in some strains after treatment with verapamil and fluconazole, meaning the inhibited yeast growth was resulting from pump inhibition. Similar outcomes for fluconazole used with Pluronic could indicate a similar mechanism for both components, i.e., Pluronic and verapamil. Next, we evaluated the main mechanism of resistance among the investigated strains. The gene expression data suggested that the expression of genes encoding efflux pumps is increased (CDR1 and CDR2). Thus, we have concluded that Pluronic might be responsible for targeting the efflux pumps and disturbing their function. This might lead to enhanced activity of fluconazole as it is not rinsed from the cell by nonfunctioning membrane transporters. If, on the contrary, the overexpression of ERG11 genes had been observed, it would have been an indication of the other mechanism of interference of Pluronic with fungal function.

In addition, we investigated the possible attenuated efflux and potential membrane permeability upon the addition of Pluronic by fluorescence microscopy. Two assays were involved, differing by the reference substance (verapamil and amphotericin B, accordingly), exhibiting various mechanisms of action (inhibition of efflux pumps and increased permeability of the fungal membranes, respectively). We observed the incorporation of Nile red, a substrate for efflux pumps, within the nontreated cells. The amount of stained cells upon incubation with Pluronic and verapamil was much lower in comparison to the treated cells, which could be explained by their disruptive effect on the membrane transporters. Propidium iodide, characterized by crossing damaged membranes, has accumulated within the cells when treated with Pluronic, however, to a lesser extent when compared to amphotericin B. In both approaches, the various amounts of stained cells (when cells were treated with Pluronic F-127 vs nontreated) indicated either impaired function of efflux pumps or attenuated integrity of the cell membrane. Therefore, the results might be interpreted as a mixed mode of action presented by the investigated polymer, resulting in enhanced activity when applied with fluconazole.

Taking into account the antifungal activity of FLU assisted by PLX presented in our study, we conclude that this block copolymer should be regarded as a valuable excipient when designing therapeutics against *Candida* infections.

Our results indicate that block copolymer itself does not cause ≥50% inhibition of yeast growth when absorbance of treated cells compared to nontreated control cells. Similarly, no inhibition zone is produced by the neat polymer. However, due to its surface-active properties, it may trigger the structural reorganization of the wall or membrane of the yeast or interrupt the activity of the efflux pumps, similarly to the mechanism reported for some types of membrane transporters.^16^ Therefore, a combination of PLX and FLU can result in synergistic antifungal activity that may arise from enhanced FLU penetration into the fungi or its retention in the cytosol.

## 4 Conclusions

Pluronic F-127-based formulations loaded with fluconazole increased the in vitro antifungal efficacy of FLU against resistant *Candida* strains. The absorbance measured in the broth microdilution method that corresponded to the yeast growth decreased significantly upon the addition of Pluronic F-127. These results agreed with the increased inhibition zone observed in the cup plate method in formulations containing this block copolymer. The highest efficacy of FLU was observed in the presence of 5.0 and 10.0% w/v of Pluronic F-127, as shown in the microbiological studies. Eight out of 15 investigated *C. glabrata* strains exhibited significant increases in the expression of genes encoding efflux pumps, and the subsequent seven tested *C. glabrata* strains displayed a higher expression level of CDR1 and CDR2 genes, indicating that Pluronic-based formulations affected the efflux mechanism. It was in line with the study of efflux pumps using verapamil that suggested that both Pluronic and verapamil might share a similar mechanism of action. The attenuated efflux and membrane integrity upon Pluronic treatment were visualized by fluorescence microscopy. Pluronic F-127 at concentrations of 20.0 and 25.0% w/v caused clear sol–gel transitions above 25 and 20 °C, respectively, in contrast to 10–15% w/v of polymer content characterized by no such phase transition. The presence of Pluronic F-127 enhanced the solubility of fluconazole, which was stable in the micellar solution upon storage for over 84 days at room temperature.

To the best of our knowledge, this is the first attempt to study the influence of Pluronic-based formulations on *Candida* growth. Not only the enhanced antifungal activity of fluconazole when assisted by block copolymer was observed, but also the potential mechanism of action of Pluronic F-127, comprising an impact on membrane transporters in fungal cells and membrane integrity, was presented in the study. Therefore, the results might facilitate the development of novel fluconazole formulations in light of the small amount of marketed fluconazole topical products.

## 5 Materials and Methods

### 5.1 Chemicals

Pluronic F-127, RPMI-1640 medium with the addition of l-glutamine and without sodium bicarbonate, PBS (phosphate-buffered saline), propidium iodide, and amphotericin B were purchased from Sigma-Aldrich (USA). Nile red was obtained from Santa Cruz Biotechnology (USA). Fluconazole and verapamil hydrochloride were obtained from Pol-Aura (Poland). Sterile, purified water was purchased from Polpharma (Poland). 3-morpholinopropanesulfonic acid (MOPS) was purchased from J&K Scientific (China). Mueller Hinton LAB-AGAR with glucose and methylene blue were both purchased from Biomaxima (Poland). Sabouraud dextrose agar with chloramphenicol was purchased from Liofilchem (Italy). Sabouraud dextrose liquid medium was purchased from Oxoid (Great Britain). Tryptic soy broth was bought from Biomaxima (Poland). Zymo Research—YeaStar RNA Kit was obtained from TK Biotech (Poland). The primers were ordered from Genomed (Poland). The TranScriba Kit and the RT HS-PCR Mix Probe were supplied by A&A Biotechnology (Poland).

### 5.2 *Candida* strains

Thirty *Candida* strains were involved in the study, including 3 from the American type culture collection (*C. krusei* ATCC 6258, *C. albicans* ATCC MYA-574, and *C. albicans* ATCC 64124)^37^ and 27 clinical isolates from the collection of the Department of Pharmaceutical Microbiology and Parasitology at Wroclaw Medical University (*C. albicans*, *C. glabrata,* and *C. tropicalis*, see detailed list in Tables 3 and 4). *C. albicans* was chosen as the most frequent cause of severe fungal infections, while *C. glabrata* was selected as frequently developing multidrug resistance as a result of its haploid genome.^38,39^ Among the investigated strains, 21 were classified as resistant to fluconazole, whereas 8 strains were identified as susceptible to increased exposure based on EUCAST Antifungal Clinical Breakpoints.^40^ In the case of *C. albicans* and *C. tropicalis*, MIC values >4 mg/L indicated resistant strains.^40^*C. glabrata* strains characterized by MIC values ≤16 mg/L were classified as susceptible to exposure, while those with MIC values >16 mg/L were classified as resistant.^40^*C. krusei* ATCC 6258 was selected as the quality control strain^40^ and is classified as naturally resistant to fluconazole. *C. albicans* ATCC MYA-574 strain overexpresses the ABC transporter genes CDR1 and CDR2 that encode ATP-dependent efflux pumps, while *C. albicans* ATCC 64124 has mutations in the ERG11 genes that affect the FLU binding to its target protein.^41^ The resistance of both strains against fluconazole has been confirmed in several reports.^42−49^ MIC values evaluated for the investigated strains were consistent with the previously published data.^50,51^ The strains were stored in tryptic soy broth with the addition of 15% glycerol and kept at −80 °C. Before experiments, strains were grown on Sabouraud dextrose agar at 35 °C for 24 h. Afterward, they were suspended in RPMI 1640 medium in double strength to a cell density of 0.5 McFarland.

### 5.3 Pluronic F-127 and Fluconazole Formulations

Pluronic F-127/fluconazole formulations were obtained by a method called “direct dissolution”, namely, dissolving the polymer in an aqueous solvent and adding the pharmaceutically active substance. The active substance is either in the form of a preprepared solution or added directly in solid form to the polymer solution.^52^

For the broth microdilution method, stock solutions of fluconazole and Pluronic F-127 were prepared in sterile purified water and then mixed in order to obtain a 2-fold concentrated solution (0.16% w/v and 10.0% w/v Pluronic F-127 loaded with 1–256 mg/L, 0.0028–0.84 mM fluconazole) added into each well of a 96-well microtiter plate (series FLU-MIC, PLX-0.08, PLX-0.08_FLU-MIC, PLX-5, and PLX-5_FLU-MIC).

For the cup plate method and rheological studies, fluconazole and Pluronic F-127 solutions were prepared in sterile purified water, while Pluronic F-127 solutions loaded with fluconazole were prepared by the addition of fluconazole solution in sterile purified water at concentrations of 4.1, 8.2, 12.2, and 14.7 mM into Pluronic F-127 powder at concentrations of 10.0, 15.0, 20.0, and 25.0% w/v and stirred until dissolved (series FLU-4–15, PLX-10, PLX-10_FLU-4–15, PLX-15, PLX-15_FLU-4–15, PLX-20, PLX-20_FLU-4–15, PLX-25, and PLX-25_FLU-4–15).

For the drug content test, 10 mg/L fluconazole exceeding the maximum solubility within 5.0% w/v of Pluronic F-127 was added to the polymer solution in sterile purified water (at concentrations of 0.08, 0.1, 0.15, 0.5, 1.0, and 5.0% w/v), and the solution was stirred at room temperature for 24 h and then filtered using a syringe filter with a 0.20 μm pore size, resulting in the series PLX-0.08_FLU-S, PLX-0.1_FLU-S, PLX-0.15_FLU-S, PLX-0.5_FLU-S, PLX-1_FLU-S, and PLX-5_FLU-S.

### 5.4 Rheological Studies

The rheological properties of Pluronic F-127 solutions (blank and loaded with fluconazole) were analyzed by the rotational rheometer Brookfield RVDV-III+ using cones CP40 and CP51 in a controlled shear rate mode.^23^ The temperature in the sample cup was controlled by a circulating water bath. For each experiment, 0.5 mL of the sample was used. An appropriate type of cone and rotational speed were selected for each of the series, taking into account their different viscosity ranges and the measurement limitations of the rheometer. The temperature of the phase transition and the temperature coefficient were evaluated based on viscosity vs temperature profiles. The spindle was rotated at 40 rpm for PLX-10 and PLX-10_FLU-4–15 formulations, at 20 rpm for PLX-15, PLX-15_FLU-4–15, and at 0.05 rpm in PLX-20, PLX-20_FLU-4–15, PLX-25, and PLX-25_FLU-4–15 formulations (shear rates equal to 300, 150, and 0.192 s^–1^, respectively). The temperature was increased at a rate of 1 °C/min in the range 20–40 °C for formulations PLX-10, PLX-10_FLU-4–15, PLX-15, PLX-15_FLU-4–15, PLX-20, and PLX-20_FLU-4–15 and in the range of 15–25 °C for formulations PLX-25 and PLX-25_FLU-4–15. Taking into account points comprising the steepest part of the plot, the equation of the linear function was established, and the phase transition was calculated for *y* = 0.

### 5.5 High-Performance Liquid Chromatography

The stability of fluconazole loaded into Pluronic F-127 solutions was analyzed according to the USP 32 monograph^53^ using the 1260 Infinity (Agilent Technologies) HPLC system equipped with a 260 nm detector and the Zorbax column SB-C18 (4.6 × 150 mm) with 5 μm packing. The column temperature was 40 °C. A mixture of water and acetonitrile (80:20) was involved as a mobile phase in isocratic elution. The flow rate was 0.5 mL per minute. The injected volume of the sample was 20 μL. The retention time of fluconazole was 7 min. Samples were stored at room temperature for 84 days and filtered using a syringe filter with a 0.20 μm pore size diameter prior to each measurement.

### 5.6 MIC Determination

MIC determination was conducted according to the European Committee on Antimicrobial Susceptibility Testing (EUCAST) standards involving the broth microdilution method.^54^ RPMI 1640 medium with the addition of l-glutamine without sodium bicarbonate buffered to pH 7.0 was prepared in double strength to allow for 1:1 dilution throughout all experiments (final concentration 0.165 M MOPS and 2% glucose).^54^ In each experiment, control tests were involved, including RPMI 1640 medium without drug, polymer, and yeasts (sterility control) and suspension of tested yeasts in RPMI 1640 medium without drug and polymer (growth control). To examine whether the addition of polymer might inhibit yeast growth, control growth wells comprised suspensions of tested yeasts in RPMI 1640 medium with block polymer (series PLX-0.08 and PLX-5). 50 μL of drug solution or drug loaded into Pluronic F-127 solution was added to one well of a 96-well microtiter plate, and then 50 μL of RPMI 1640 medium with yeasts was placed. After inoculation, plates were incubated for 24 h at 35 °C. Absorbance within wells was measured at 530 nm using a MultiScan Go Spectrophotometer (Thermo Fisher Scientific). The MIC values of fluconazole alone and of all combinations were read as the lowest concentration that caused growth inhibition by ≥50% in comparison to nontreated control cells. The final concentration of fluconazole in each well was in the range of 0.5–16 mg/L (0.0016–0.052 mM for susceptible *Candida* strains) and 4–128 mg/L (0.013–0.42 mM for resistant *Candida* strains) comprising the FLU-MIC series, whereas the final concentration of block polymer in the micellar series throughout all the wells was 0.08% w/v (series PLX-0.08_FLU-MIC) and 5.0% w/v (series PLX-5_FLU-MIC), respectively. The final concentration of yeasts in each well was approximately 1.5 × 10^5^ cfu/mL. Every experiment was conducted at least three times.

### 5.7 Investigation of Efflux Pumps with Verapamil

Verapamil is known as an inhibitor of ABC efflux pumps in the cell membranes of fungi. Therefore, the MIC determination assay was also performed in its presence to observe any potential changes in MIC values upon the addition of verapamil. Stock solutions of verapamil hydrochloride in sterile, purified water were prepared. It was added to the suspension of tested yeasts in RPMI 1640 medium with fluconazole, and the final concentration of 100 μM of verapamil in a well was obtained (series VER_FLU-MIC) according to the protocol described in section “MIC Determination”. To verify whether neat verapamil might inhibit yeast growth, control growth wells comprised of suspensions of tested yeasts in RPMI 1640 medium with verapamil (series VER) were used. A decrease in the MIC value due to the presence of verapamil may indicate the resistance mechanism correlated to overexpression of ABC and/or MDR efflux pumps.^55−57^

### 5.8 Cup Plate Method

The antifungal activity of Pluronic F-127 at 10–25% w/v concentrations loaded with fluconazole was investigated by the cup plate method. The density of *Candida* cells inoculated on the surface of Mueller Hinton agar with the addition of glucose and methylene blue was 0.5 McFarland. Fourteen strains resistant to fluconazole and *C. krusei* ATCC 6258 were involved in the study. Wells with 7 mm diameter were cut with a sterile cork borer in the medium. 20 μL of fluconazole solution (series FLU-4–15) or Pluronic F-127 solutions loaded with fluconazole (series PLX-10_FLU-4–15, PLX-15_FLU-4–15, PLX-20_FLU-4–15, PLX-25_FLU-4–15) was placed in each well on an agar plate. The final concentrations of fluconazole in each well were 25, 50, 75, and 90 μg. To investigate whether the addition of polymer might inhibit yeast growth, subsequent wells for control growth were filled with Pluronic F-127 solutions without the addition of fluconazole (PLX-10, PLX-15, PLX-20, and PLX-25). After inoculation, plates were incubated for 24 h at 35 °C. The diameter of the zone of growth inhibition was measured. Every experiment was conducted at least three times.

### 5.9 Studies on the Kinetics of Antifungal Activity

Studies on the kinetics of the antifungal activity were performed for the formulations with the best outcomes evaluated based on results from broth microdilution and cup plate methods (with a content of 5% w/v and 10% w/v of Pluronic F-127, series FLU-MIC, PLX-5_FLU-MIC, and PLX-10_FLU-MIC, respectively) according to the procedure described in the section “MIC Determination”. The growth kinetics was investigated for the representative strains, *C. krusei* ATCC 6258, *C. albicans* 3057, *C. glabrata* 2586, *C. glabrata* 2738, and *C. glabrata* 2853. The absorbance mode was set for the reading every 20 min at 530 nm under constant shaking. It was programmed to take seventy-three individual measurements in total over a 24 h period.

### 5.10 Microscopic Imaging in Phase Contrast

Particular wells with cultivated fungi in the presence and absence of fluconazole after 24 h at 35 °C on the 96-well microtiter plates were observed in phase contrast using a fluorescence microscope, an Olympus IX53. Final concentration of Pluronic F-127 in the studied wells was 0.08% and 5.0% w/v. Images were acquired with the Olympus cellSens imaging software.

### 5.11 Imaging by Fluorescence Microscopy

Cellular efflux of *Candida* species and the potential permeability of the fungal membranes were examined based on the method described by Iyer et al. with slight modifications.^25^*C. albicans* 3057, *C. glabrata2586*, *C. glabrata2738*, and *C. glabrata2853* were selected as representatives for the study. Yeasts were cultured on Sabouraud agar plates at 35 °C for 24 h. Thereafter, they were suspended in a Sabouraud broth and incubated at 35 °C overnight with constant shaking. The density of all strains was diluted in Sabouraud broth to an OD_600_ value of 0.02 in 1 mL (in order to obtain the final density of 0.01 that corresponds to the yeasts density in each well in broth microdilution method, see section “MIC Determination”). Afterward, strains were cultured with agitation at 35 °C for ca. 3–4 h until the exponential phase was reached.

In the experiment assessing the cellular efflux, three subcultures were set for each strain, i.e., stained, stained in the presence of a reference efflux inhibitor, verapamil, and stained in the presence of a studied substance (Pluronic F-127). Either the compound solution or sterile water (in the control subculture) was added to yeast suspension to allow for 1:1 dilution and incubated for either 20 min (verapamil) or 24 h (Pluronic F-127) at 35 °C under constant agitation. The final concentration of verapamil was 100 μM, and 10% w/v of Pluronic F-127. A stock solution of Nile red in DMSO at a concentration of 3.5 mM was added to each of the incubated samples to a final concentration of 7 μM and incubated for 20 min at 35 °C under constant agitation. Each subculture was transferred to an Eppendorf tube and centrifuged for 1 min at 14,000 rpm. The supernatant was removed, and the pellet was resuspended in 100 μL of PBS.

In the experiment assessing the potential permeability of the fungal membranes, three subcultures were set for each strain, i.e., stained, stained in the presence of a reference substance, amphotericin B, and stained in the presence of a studied substance (Pluronic F-127). Either the compound solution or sterile water (in the control subculture) was added to the yeast suspension to allow for 1:1 dilution and incubated for either 1 h (amphotericin B) or 24 h (Pluronic F-127) at 35 °C under constant agitation. The final concentration of amphotericin B was 2 μg/mL, and 10% w/v of Pluronic F-127. Each subculture was transferred to an Eppendorf tube and centrifuged for 1 min at 14,000 rpm. The supernatant was removed, and the pellet was resuspended in 100 μL of PBS. The cells were treated with propidium iodide prior to the imaging, and the final concentration amounted to 1 μM.

A few μL of Candida suspension in PBS was placed on a glass slide and covered with a coverslip. Visualization was performed using a 40× objective lens on the TRITC channel (excitation 544 nm, emission 570 nm) by a fluorescence microscope, an Olympus IX53. Images were acquired with Olympus cellSens imaging software.

### 5.12 Gene Expression Analysis

Gene expression analysis was performed based on the method described by Szweda et al. with slight modifications.^58^ Yeasts were cultured on Sabouraud agar plates at 35 °C for 24 h. Two single colonies of each of the investigated strains were suspended in 4 mL of Sabouraud broth and incubated at 35 °C with constant shaking until an OD_660_ value in the range of 1.5–1.9 was obtained. The density of all strains was diluted to a similar OD_660_ of approximately 1.4, which corresponds to 3.8 × 10^7^ cells. RNA was isolated using the YeaStar RNA Kit. In the first step, yeast cells were centrifuged at 500 g for 2 min. 80 μL YR Digestion Buffer and 5 μL Zymolyase were added after the removal of the supernatant.

The suspension was incubated at 35 °C for 1 h, and then 160 μL of YR Lysis Buffer was added, followed by the addition of 245 μL of ethanol 96%. The mixture was transferred to the Zymo-Spin IIICG Column, centrifuged at 15,000*g* for 30 s, and afterward, flow-through was discarded. The same procedure was repeated twice with 200 μL of RNA wash buffer. In the next step, the column was transferred into a nuclease-free tube, and 60 μL of DNase/RNase-Free Water was added and centrifuged at 15,000*g* for 30 s. The concentration and purity of RNA were determined by NanoDrop2000C (Thermo Scientific). A reverse transcription reaction was performed with a TranScriba Kit. First, 10 μL of the mixture containing 140 ng of isolated RNA, 1 μL of oligo(dT)_18_, and DNase/RNase-Free Water was incubated at 65 °C for 5 min. Afterward, 4 μL of 5× reaction buffer, 2 μL of dNTP’s mix, and 4 μL of TranScriba reverse transcriptase were added, and the mixture was incubated at 42 °C for 60 min. The reaction was terminated by temperature enhancement up to 70 °C for 5 min. The concentration and purity of cDNA were determined by NanoDrop2000C (Thermo Scientific). Analysis of the level expression of target genes ERG11, CDR1, CDR2, and a reference gene URA3 was performed by real-time PCR using the Toptical Gradient 96 Real-Time PCR system (Biometra). Primers and probes were chosen as previously described^58,59^ (Supporting Information, Table S1), and solutions in buffer TE at concentrations of 10 μM were prepared.

The mixture for each real-time PCR reaction consisted of 10 μL of the 2× Real Time HS-PCR Master Mix Probe, 0.5 μM each primer solution, 0.25 μM probe solution, and 1 μL of cDNA filled with DNase/RNase-Free Water to the final volume of 20 μL. A 2-fold serial dilution of the pooled cDNA mixture of all investigated strains was used for standard curves, whereas 7 ng from cDNA of each of the studied strains was involved for gene expression analysis. Primer efficiency tests for each of the investigated genes were run in triplicate, while experiments for the evaluation of the level of expression of the studied genes were run in duplicate. The following conditions for amplification were set: initial denaturation at 95 °C for 5 min, 50 cycles of denaturation at 95 °C for 15 s, primer annealing at 59, 59, 62, and 55 °C (respectively for ERG11, CDR1, CDR2, and URA3) for 15 s, and elongation step at 72 °C for 15 s. The temperature transition rate was defined as 20 °C/s.

### 5.13 Statistical Analysis

The results obtained in the experiments were variables on interval and quotient scales. The statistical methods used in the analysis were simple linear regression, nonlinear estimation, and tests to compare means. All results subjected to the statistical analyses performed were mean values (MV) calculated from 6 independent replicates. The precision of the obtained averages was evaluated by determining for each mean two descriptive statistics recommended by Pharmacopoeia XII, namely, standard deviation (SD) and relative standard deviation (RSD = SD/MV). A parametric MANOVA with a posthoc test for multiple comparisons (Fisher’s Least Significant Difference test) was used to evaluate differences between MV means. All variables compared using the MANOVA test met the assumptions of normality of distribution and homogeneity of variance. The normality of the distributions of the compared variables was tested with three different statistical tests: the Kolmogorov–Smirnov test, the Lillefors test, and the W. Shapiro–Wilk test. Homogeneity of variance was assessed with the Brown–Forsyth and Levene’s tests. Simple linear regression and nonlinear estimation were used to determine correlations between variables. In both cases, the loss function was minimized using the method of least squares. The statistical significance of the performed estimations was assessed by determining Pearson’s *r*^2^ correlation coefficients for linear models and *R*-determination coefficients for nonlinear ones, the statistical significance of which was evaluated by the *t*-test.

The overall relationships between all the statistically evaluated variables were analyzed and then visualized using multivariate analyses based on dimension reduction using the procedure of decomposing the matrix of results according to singular values: principal component analysis (PCA) and correspondence analysis. The constructed PCA models were estimated using the NIPALS iterative algorithm, with the convergence criterion set at 0.00001 and the maximum number of iterations equal to 100. The number of principal components was determined by determining the maximum predictive ability of *Q*^2^ using the *V*-fold cross-check method, setting the maximum number of them at *V*_max_ = 7. The obtained optimal PCA model was graphically visualized in the graph of the two principal components, characterized by the largest percentage contribution to the variance explained by the model (PC1 vs PC2) reduced to two components. The results of the PCA analysis shown in the PC1 and PC2 load graphs made it possible to preselect the variables having the most significant impact on the model built and to select the most significant relationships between them. The variables selected in this way were then subjected to further statistical evaluation.

In all the statistical analyses, the level of significance was adopted as α = 0.05. The statistical analyses were performed using STATISTICA PL 13.3 and Mathematica 10.0.

Livak’s method was involved in the analysis of the level of gene expression.^24^ The method enabled us to establish differences between the expression levels of target genes in the investigated strains and a reference strain. In our study, *C. glabrata* 1004 from the collection of the Department of Pharmaceutical Microbiology and Parasitology at Wroclaw Medical University with the MIC value for fluconazole equal to 8 mg/L was designated as a reference strain. The level of gene expression of target genes was assessed by comparing the threshold cycle values (*C*_t_) of the amplification of a target gene and a reference gene. If the 2^–ΔΔ*CT*^ value amounts to 1, it means that the expression level of a target gene in the investigated and reference strains is the same. The 2^–ΔΔ*CT*^ value in the range between 1 and 2 indicates a higher expression level of a target gene in the investigated strain in comparison to a reference strain, while the value above 2 stands for a significant enhancement of the expression level of the target gene with regard to a reference strain.

## Supporting Information Available

Results from rheological and high-performance liquid chromatography studies, statistical analysis, microscopic imaging, raw data from the cup plate method, and expression levels of investigated genes (PDF)

## References

[ref1] Perlin D. S.; Rautemaa-Richardson R.; Alastruey-Izquierdo A. The Global Problem of Antifungal Resistance: Prevalence, Mechanisms, and Management. Lancet Infect. Dis. 2017, 17 (12), e383–e392. 10.1016/S1473-3099(17)30316-X. 28774698

[ref2] Benedict K.; Jackson B. R.; Chiller T.; Beer K. D. Estimation of Direct Healthcare Costs of Fungal Diseases in the United States. Clin. Infect. Dis. 2019, 68 (11), 1791–1797. 10.1093/cid/ciy776. 30204844 PMC6409199

[ref3] Denning D. W.; Kneale M.; Sobel J. D.; Rautemaa-Richardson R. Global Burden of Recurrent Vulvovaginal Candidiasis: A Systematic Review. Lancet Infect. Dis. 2018, 18 (11), e339–e347. 10.1016/S1473-3099(18)30103-8. 30078662

[ref4] Lírio J.; Giraldo P. C.; Amaral R. L.; Sarmento A. C. A.; Costa A. P. F.; Goncalves A. K. Antifungal (Oral and Vaginal) Therapy for Recurrent Vulvovaginal Candidiasis: A Systematic Review Protocol. BMJ Open 2019, 9 (5), e027489 10.1136/bmjopen-2018-027489. PMC6537984 31122991

[ref5] Gonçalves B.; Ferreira C.; Alves C. T.; Henriques M.; Azeredo J.; Silva S. Vulvovaginal Candidiasis: Epidemiology, Microbiology and Risk Factors. Crit. Rev. Microbiol. 2016, 42 (6), 905–927. 10.3109/1040841X.2015.1091805. 26690853

[ref6] Rahme D.; Ayoub M.; Shaito K.; Saleh N.; Assaf S.; Lahoud N. First Trend Analysis of Antifungals Consumption in Lebanon Using the World Health Organization Collaborating Center for Drug Statistics Methodology. BMC Infect. Dis. 2022, 22 (1), 882 10.1186/s12879-022-07883-5. 36434539 PMC9700908

[ref7] Brown G. D.; Denning D. W.; Gow N. A. R.; Levitz S. M.; Netea M. G.; White T. C. Hidden Killers: Human Fungal Infections. Sci. Transl. Med. 2012, 4 (165), 165rv13 10.1126/scitranslmed.3004404. 23253612

[ref8] Cannon R. D.; Lamping E.; Holmes A. R.; Niimi K.; Baret P. V.; Keniya M. V.; Tanabe K.; Niimi M.; Goffeau A.; Monk B. C. Efflux-Mediated Antifungal Drug Resistance. Clin. Microbiol. Rev. 2009, 22 (2), 291–321. 10.1128/CMR.00051-08. 19366916 PMC2668233

[ref9] Warrilow A. G.; Parker J. E.; Kelly D. E.; Kelly S. L. Azole Affinity of Sterol 14-Demethylase (CYP51) Enzymes from Candida Albicans and Homo Sapiens. Antimicrob. Agents Chemother. 2013, 57 (3), 1352–1360. 10.1128/AAC.02067-12. 23274672 PMC3591892

[ref10] European Medicine Agency. List of Nationally Authorised Medicinal Products (Fluconazole). Procedure No.: PSUSA/00001404/201703, 2017. https://www.ema.europa.eu/en/documents/psusa/fluconazole-list-nationally-authorised-medicinal-products-psusa/00001404/201703_en.pdf.

[ref11] Nowak M.; Dyba A. J.; Janczak J.; Morritt A.; Fábián L.; Karolewicz B.; Khimyak Y. Z.; Braun D. E.; Nartowski K. P. Directing Crystallization Outcomes of Conformationally Flexible Molecules: Polymorphs, Solvates, and Desolvation Pathways of Fluconazole. Mol. Pharmaceutics 2022, 19, 456–471. 10.1021/acs.molpharmaceut.1c00752. 35050637

[ref12] Redliński A.; Czekaj T.; Ciszewski M. Off Label Use Cases and Orphan Drugs in Pharmaceutical Practice. Part 1. Liquid Drug Forms. Farm. Polym. 2012, 68 (4), 219–227.

[ref13] Bernardes M.; Hohl T. M. Fungal Infections Associated With the Use of Novel Immunotherapeutic Agents. Curr. Clin. Microbiol. Rep. 2020, 7 (4), 142–149. 10.1007/s40588-020-00154-4. 34336548 PMC8320460

[ref14] Armstrong-James D.; Brown G. D.; Netea M. G.; Zelante T.; Gresnigt M. S.; van de Veerdonk F. L.; Levitz S. M. Immunotherapeutic Approaches to Treatment of Fungal Diseases. Lancet Infect. Dis. 2017, 17 (12), e393–e402. 10.1016/S1473-3099(17)30442-5. 28774700

[ref15] Karolewicz B. A Review of Polymers as Multifunctional Excipients in Drug Dosage Form Technology. Saudi Pharm. J. 2016, 24 (5), 525–536. 10.1016/j.jsps.2015.02.025. 27752224 PMC5059828

[ref16] Alakhova D. Y.; Kabanov A. V. Pluronics and MDR Reversal: An Update. Mol. Pharmaceutics 2014, 11, 2566–2578. 10.1021/mp500298q. PMC4122590 24950236

[ref17] Pathadka S.; Yan V. K. C.; Neoh C. F.; Al-Badriyeh D.; Kong D. C. M.; Slavin M. A.; Cowling B. J.; Hung I. F. N.; Wong I. C. K.; Chan E. W. Global Consumption Trend of Antifungal Agents in Humans From 2008 to 2018: Data From 65 Middle- and High-Income Countries. Drugs 2022, 82 (11), 1193–1205. 10.1007/s40265-022-01751-x. 35960433 PMC9402496

[ref18] White T. C.; Marr K. A.; Bowden R. A. Clinical, cellular, and molecular factors that contribute to antifungal drug resistance. Clin. Microbiol. Rev. 1998, 11 (2), 382–402. 10.1128/CMR.11.2.382. 9564569 PMC106838

[ref19] Liu S.; Bao H.; Li L. Role of PPO-PEO-PPO Triblock Copolymers in Phase Transitions of a PEO-PPO-PEO Triblock Copolymer in Aqueous Solution. Eur. Polym. J. 2015, 71, 423–439. 10.1016/j.eurpolymj.2015.08.016.

[ref20] Malec K.; Monaco S.; Delso I.; Nestorowicz J.; Kozakiewicz-Latała M.; Karolewicz B.; Khimyak Y. Z.; Angulo J.; Nartowski K. P. Unravelling the Mechanisms of Drugs Partitioning Phenomena in Micellar Systems via NMR Spectroscopy. J. Colloid Interface Sci. 2023, 638, 135–148. 10.1016/j.jcis.2023.01.063. 36736115

[ref21] Chroni A.; Chrysostomou V.; Skandalis A.; Pispas S. Drug Delivery: Hydrophobic Drug Encapsulation into Amphiphilic Block Copolymer Micelles. Supramolecules in Drug Discovery and Drug Delivery; Methods in Molecular Biology; Springer, 2021; Vol. 2207, pp 71–83. 10.1007/978-1-0716-0920-0_6 33113128

[ref22] Irwan A. W.; Berania J. E.; Liu X. A Comparative Study on the Effects of Amphiphilic and Hydrophilic Polymers on the Release Profiles of a Poorly Water-Soluble Drug. Pharm. Dev. Technol. 2016, 21 (2), 231–238. 10.3109/10837450.2014.991877. 25496001

[ref23] Burak J.; Grela K. P.; Pluta J.; Karolewicz B.; Marciniak D. M. Impact of Sterilisation Conditions on the Rheological Properties of Thermoresponsive Pluronic F-127-Based Gels for the Ophthalmic Use. Acta Polym. Pharm.—Drug Res. 2018, 75 (2), 471–481. 10.32383/appdr/146102.

[ref24] Livak K. J.; Schmittgen T. D. Analysis of Relative Gene Expression Data Using Real-Time Quantitative PCR and the 2-ΔΔCT Method. Methods 2001, 25 (4), 402–408. 10.1006/meth.2001.1262. 11846609

[ref25] Iyer K. R.; Robbins N.; Cowen L. E. Flow Cytometric Measurement of Efflux in Candida Species. Curr. Protoc. Microbiol. 2020, 59 (1), e121 10.1002/cpmc.121. 33047867 PMC8515609

[ref26] Holmes A. R.; Cardno T. S.; Strouse J. J.; Ivnitski-Steele I.; Keniya M. V.; Lackovic K.; Monk B. C.; Sklar L. A.; Cannon R. D. Targeting Efflux Pumps to Overcome Antifungal Drug Resistance. Future Med. Chem. 2016, 8 (12), 1485–1501. 10.4155/fmc-2016-0050. 27463566 PMC5827819

[ref27] Crowley L. C.; Scott A. P.; Marfell B. J.; Boughaba J. A.; Chojnowski G.; Waterhouse N. J. Measuring Cell Death by Propidium Iodide Uptake and Flow Cytometry. Cold Spring Harb. Protoc. 2016, 7, 647–651. 10.1101/pdb.prot087163. 27371595

[ref28] Fernández-García R.; Muñoz-García J. C.; Wallace M.; Fabian L.; González-Burgos E.; Gómez-Serranillos M. P.; Raposo R.; Bolás-Fernández F.; Ballesteros M. P.; Healy A. M.; Khimyak Y. Z.; Serrano D. R. Self-Assembling, Supramolecular Chemistry and Pharmacology of Amphotericin B: Poly-Aggregates, Oligomers and Monomers. J. Controlled Release 2022, 341, 716–732. 10.1016/j.jconrel.2021.12.019. 34933052

[ref29] Lo W. H.; Deng F. S.; Chang C. J.; Lin C. H. Synergistic Antifungal Activity of Chitosan with Fluconazole against Candida Albicans, Candida Tropicalis, and Fluconazole-Resistant Strains. Molecules 2020, 25 (21), 5114 10.3390/molecules25215114. 33153228 PMC7663520

[ref30] Grimling B.; Karolewicz B.; Nawrot U.; Włodarczyk K.; Górniak A. Physicochemical and Antifungal Properties of Clotrimazole in Combination with High-Molecular Weight Chitosan as a Multifunctional Excipient. Mar. Drugs 2020, 18 (12), 591 10.3390/md18120591. 33255899 PMC7760713

[ref31] Shih P. Y.; Liao Y. T.; Tseng Y. K.; Deng F. S.; Lin C. H. A Potential Antifungal Effect of Chitosan against Candida Albicansis Mediated via the Inhibition of SAGA Complex Component Expression and the Subsequent Alteration of Cell Surface Integrity. Front. Microbiol. 2019, 10, 602 10.3389/fmicb.2019.00602. 30972050 PMC6443709

[ref32] Batrakova E. V.; Kabanov A. V. Pluronic Block Copolymers: Evolution of Drug Delivery Concept from Inert Nanocarriers to Biological Response Modifiers. J. Controlled Release 2008, 130 (2), 98–106. 10.1016/j.jconrel.2008.04.013. PMC2678942 18534704

[ref33] Alburquenque C.; Bucarey S. A.; Neira-Carrillo A.; Urzúa B.; Hermosilla G.; Tapia C. V. Antifungal Activity of Low Molecular Weight Chitosan against Clinical Isolates of Candida Spp. Med. Mycol. 2010, 48 (8), 1018–1023. 10.3109/13693786.2010.486412. 20482450

[ref34] Garcia L. G. S.; Guedes G. M. d. M.; da Silva M. L. Q.; Castelo-Branco D. S. C. M.; Sidrim J. J. C.; Cordeiro R. d. A.; Rocha M. F. G.; Vieira R. S.; Brilhante R. S. N. Effect of the Molecular Weight of Chitosan on Its Antifungal Activity against Candida Spp. in Planktonic Cells and Biofilm. Carbohydr. Polym. 2018, 195, 662–669. 10.1016/j.carbpol.2018.04.091. 29805025

[ref35] Ardizzoni A.; Neglia R. G.; Baschieri M. C.; Cermelli C.; Caratozzolo M.; Righi E.; Palmieri B.; Blasi E. Influence of Hyaluronic Acid on Bacterial and Fungal Species, Including Clinically Relevant Opportunistic Pathogens. J. Mater. Sci.: Mater. Med. 2011, 22 (10), 2329–2338. 10.1007/s10856-011-4408-2. 21892787

[ref36] Kang J. H.; Kim Y. Y.; Chang J. Y.; Kho H. S. Influences of Hyaluronic Acid on the Anticandidal Activities of Lysozyme and the Peroxidase System. Oral Dis. 2011, 17 (6), 577–583. 10.1111/j.1601-0825.2011.01807.x. 21477181

[ref37] American Type Culture Collection. Multidrug-Resistant and Antimicrobial Testing Reference Strains, 2019. https://www.summitpharma.co.jp/japanese/service/pdf/Multidrug-Resistant%20and%20Antimicrobial%20Testing%20Reference%20Strains.pdf.

[ref38] Toda M.; Williams S. R.; Berkow E. L.; Farley M. M.; Harrison L. H.; Bonner L.; Marceaux K. M.; Hollick R.; Zhang A. Y.; Schaffner W.; Lockhart S. R.; Jackson B. R.; Vallabhaneni S. Population-Based Active Surveillance for Culture-Confirmed Candidemia - Four Sites, United States, 2012–2016. MMWR Surveill. Summ. 2019, 68 (8), 1–15. 10.15585/mmwr.ss6808a1. PMC6772189 31557145

[ref39] Farmakiotis D.; Kontoyiannis D. P. Epidemiology of Antifungal Resistance in Human Pathogenic Yeasts: Current Viewpoint and Practical Recommendations for Management. Int. J. Antimicrob. Agents 2017, 50 (3), 318–324. 10.1016/j.ijantimicag.2017.05.019. 28669831

[ref40] EUCAST. European Committee on Antimicrobial Susceptibility Testing. Breakpoint Tables for Interpretation of MICs for Antifungal Agents, Version 10.0, 2020. http://www.eucast.org/fileadmin/src/media/PDFs/EUCAST_files/Breakpoint_tables/v_5.0_Breakpoint_Table_01.pdf.

[ref41] Calabrese E. C.; Castellano S.; Santoriello M.; Sgherri C.; Quartacci M. F.; Calucci L.; Warrilow A. G. S.; Lamb D. C.; Kelly S. L.; Milite C.; Granata I.; Sbardella G.; Stefancich G.; Maresca B.; Porta A. Antifungal Activity of Azole Compounds CPA18 and CPA109 against Azole-Susceptible and -Resistant Strains of Candida Albicans. J. Antimicrob. Chemother. 2013, 68 (5), 1111–1119. 10.1093/jac/dks506. 23292344

[ref42] Mohammad H.; Eldesouky H. E.; Hazbun T.; Mayhoub A. S.; Seleem M. N. Identification of a Phenylthiazole Small Molecule with Dual Antifungal and Antibiofilm Activity Against Candida Albicans and Candida Auris. Sci. Rep. 2019, 9 (1), 18941 10.1038/s41598-019-55379-1. 31831822 PMC6908612

[ref43] Thamban Chandrika N.; Shrestha S. K.; Ranjan N.; Sharma A.; Arya D. P.; Garneau-Tsodikova S. New Application of Neomycin B-Bisbenzimidazole Hybrids as Antifungal Agents. ACS Infect. Dis. 2018, 4 (2), 196–207. 10.1021/acsinfecdis.7b00254. 29227087 PMC5971066

[ref44] Shrestha S. K.; Grilley M.; Anderson T.; Dhiman C.; Oblad J.; Chang C. W. T.; Sorensen K. N.; Takemoto J. Y. In Vitro Antifungal Synergy between Amphiphilic Aminoglycoside K20 and Azoles against Candida Species and Cryptococcus Neoformans. Med. Mycol. 2015, 53 (8), 837–844. 10.1093/mmy/myv063. 26260746

[ref45] Shrestha S. K.; Fosso M. Y.; Garneau-Tsodikova S. A Combination Approach to Treating Fungal Infections. Sci. Rep. 2015, 5, 17070 10.1038/srep17070. 26594050 PMC4655404

[ref46] Hata M.; Yoshida K.; Ishii C.; Otani T.; Ando A. In Vitro and in Vivo Antifungal Activities of Aminopiperidine Derivatives, Novel Ergosterol Synthesis Inhibitors. Biol. Pharm. Bull. 2010, 33 (3), 473–476. 10.1248/bpb.33.473. 20190412

[ref47] Kitamura A.; Someya K.; Okumura R.; Hata M.; Takeshita H.; Nakajima R. In Vitro Antifungal Activities of D11-2040, a .BETA.-1,6-Glucan Inhibitor, with or without Currently Available Antifungal Drugs. Biol. Pharm. Bull. 2010, 33 (2), 192–197. 10.1248/bpb.33.192. 20118539

[ref48] Mitsuyama J.; Nomura N.; Hashimoto K.; Yamada E.; Nishikawa H.; Kaeriyama M.; Kimura A.; Todo Y.; Narita H. In Vitro and in Vivo Antifungal Activities of T-2307, a Novel Arylamidine. Antimicrob. Agents Chemother. 2008, 52 (4), 1318–1324. 10.1128/AAC.01159-07. 18227186 PMC2292552

[ref49] Basso V.; Garcia A.; Tran D. Q.; Schaal J. B.; Tran P.; Ngole D. A.; Aqeel Y.; Tongaonkar P.; Ouellette A. J.; Selsted M. Fungicidal Potency and Mechanisms of θ-Defensins against Multidrug-Resistant Candida Species. Antimicrob. Agents Chemother. 2018, 62 (6), e00111-18 10.1128/AAC.00111-18. 29610196 PMC5971616

[ref50] Król J.; Nawrot U.; Bartoszewicz M. Anti-Candidal Activity of Selected Analgesic Drugs Used Alone and in Combination with Fluconazole, Itraconazole, Voriconazole, Posaconazole and Isavuconazole. J. Mycol. Med. 2018, 28 (2), 327–331. 10.1016/j.mycmed.2018.03.002. 29605543

[ref51] Król J.; Nawrot U.; Bartoszewicz M. Activity of Base Analogues (5-Fluorouracil, 5-Flucytosine) against Planktonic Cells and Mature Biofilm of Candida Yeast. Effect of Combination with Folinic Acid. J. Mycol. Med. 2019, 29 (2), 147–153. 10.1016/j.mycmed.2019.04.003. 31023592

[ref52] Cholkar K.; Patel A.; Dutt Vadlapudi A.; K Mitra A. Novel Nanomicellar Formulation Approaches for Anterior and Posterior Segment Ocular Drug Delivery. Recent Pat. Nanomed. 2012, 2 (2), 82–95. 10.2174/1877912311202020082. 25400717 PMC4232191

[ref53] United States Pharmacopeial Convention. The United States Pharmacopeia: USP 32; The National Formulary: NF 27, 2009; pp 2383–2384.

[ref54] Arendrup M. C.; Meletiadis J.; Mouton J. W.; Lagrou K.; Hamal P.; Guinea J.; Subcommittee on Antifungal Susceptibility Testing (AFST) of the ESCMID European Committee for Antimicrobial Susceptibility Testing (EUCAST). EUCAST EDEF 7.3.2. Method for the Determination of Broth Dilution Minimum Inhibitory Concentrations of Antifungal Agents for Yeasts, 2020. https://www.eucast.org/fileadmin/src/media/PDFs/EUCAST_files/AFST/Files/EUCAST_E_Def_7.3.2_Yeast_testing_definitive_revised_2020.pdf.

[ref55] Domingues Bianchin M.; Borowicz S. M.; da Rosa Monte Machado G.; Pippi B.; Stanisçuaski Guterres S.; Raffin Pohlmann A.; Meneghello Fuentefria A.; Clemes Külkamp-Guerreiro I. Lipid Core Nanoparticles as a Broad Strategy to Reverse Fluconazole Resistance in Multiple Candida Species. Colloids Surf., B 2019, 175, 523–529. 10.1016/j.colsurfb.2018.12.011. 30579053

[ref56] Pippi B.; Lana A. J. D.; Moraes R. C.; Güez C.; Machado M.; de Oliveira L. F. S.; Lino von Poser G.; Fuentefria A. M. In Vitro Evaluation of the Acquisition of Resistance, Antifungal Activity and Synergism of Brazilian Red Propolis with Antifungal Drugs on Candida Spp. J. Appl. Microbiol. 2015, 118 (4), 839–850. 10.1111/jam.12746. 25565139

[ref57] Pinto E Silva A. T.; Costa-De-Oliveira S.; Silva-Dias A.; Pina-Vaz C.; Rodrigues A. G. Dynamics of in Vitro Acquisition of Resistance by Candida Parapsilosis to Different Azoles. FEMS Yeast Res. 2009, 9 (4), 626–633. 10.1111/j.1567-1364.2009.00508.x. 19385998

[ref58] Szweda P.; Gucwa K.; Romanowska E.; Dzierżanowska-Fangrat K.; Naumiuk Ł.; Brillowska-Da browska A.; Wojciechowska-Koszko I.; Milewski S. Mechanisms of Azole Resistance among Clinical Isolates of Candida Glabrata in Poland. J. Med. Microbiol. 2015, 64 (6), 610–619. 10.1099/jmm.0.000062. 25818698

[ref59] Sanguinetti M.; Posteraro B.; Fiori B.; Ranno S.; Torelli R.; Fadda G. Mechanisms of Azole Resistance in Clinical Isolates of Candida Glabrata Collected during a Hospital Survey of Antifungal Resistance. Antimicrob. Agents Chemother. 2005, 49 (2), 668–679. 10.1128/AAC.49.2.668-679.2005. 15673750 PMC547307

