# Supplementary material for: Pluronic F-127 Enhances the Antifungal Activity of Fluconazole against Resistant Candida Strains
Source: ACS Infect Dis. 2023 Dec 18;10(1):215–31. doi: 10.1021/acsinfecdis.3c00536 (PMC10795414; doi:10.1021/acsinfecdis.3c00536)
Supplement: Supplementary file 1 — id3c00536_si_001.pdf [file id3c00536_si_001.pdf]

SUPPORTING INFORMATION

Pluronic® F-127 enhances antifungal activity of fluconazole against resistant *Candida* strains

Katarzyna Malec[1*], Aleksandra Mikołajczyk[2], Dominik Marciniak[1], Agnieszka Gawin-Mikołajewicz[1], Agnieszka Matera-Witkiewicz[2], Bożena Karolewicz[1], Urszula Nawrot[3], Yaroslav Z. Khimyak[1,4*], †Karol P. Nartowski[1,4]

[1]Department of Drug Form Technology, Faculty of Pharmacy, Wroclaw Medical University, 211a Borowska Str. 50-556 Wroclaw, Poland

[2]Screening Biological Activity Assays and Collection of Biological Material Laboratory, Wroclaw Medical University, 211a Borowska Str. 50-556 Wroclaw, Poland

[3]Department of Pharmaceutical Microbiology and Parasitology, Wroclaw Medical University, 211a Borowska Str. 50-556 Wroclaw, Poland

[4]School of Pharmacy, University of East Anglia, Chancellors Drive, NR4 7TJ Norwich, United Kingdom

†deceased author

*all correspondence should be addressed to:

Katarzyna Malec - k.malec@umw.edu.pl

Yaroslav Z. Khimyak - Y.Khimyak@uea.ac.uk

Number of pages: 21

Number of Figures: 14

Number of Tables: 12

Sections

1. Materials and methods 3
2. Rheological studies 4
3. Drug content in micellar solutions of Pluronic® F-127 5
4. The statistical analysis of the results of broth microdilution method performed on resistant *Candida glabrata* strains 7
5. The statistical analysis of the results of broth microdilution method performed on all examined resistant *Candida* strains (*C. krusei, C. albicans, C. glabrata, C. tropicalis*) 8
6. The statistical analysis of the results of broth microdilution method performed on susceptible *Candida glabrata* strains 10
7. Cup plate method results 11
8. The statistical analysis of the results of cup plate method performed on resistant *Candida glabrata* strains 12
9. The statistical analysis of the results of cup plate method performed on all examined resistant *Candida* strains (*C. krusei, C. albicans, C. glabrata*) 13
10. Kinetics of antifungal activity 14
11. Microscopic imaging 15
12. Gene expression analysis 18
13. References 21

1. Materials and methods

The investigated Pluronic concentrations were chosen to cover a variety of compositions differing from each other in terms of their rheological properties. The lowest investigated concentration was slightly above critical micellization concentration, CMC (0.08 % w/v)[1] to ensure the presence of micellar aggregates. The subsequent concentrations (up to 15 % w/v) were characterized by no sol-gel transition in contrary to samples with 20-25 % w/v Pluronic content. The highest investigated concentration (25.0 % w/v) was the only one that took the form of gel at room temperature.

Fluconazole concentration in samples that were investigated in terms of the stability of drug was chosen as maximum solubility at particular polymer concentration in order to provide the least favorable conditions in which the drug can precipitate. Therefore, the results can be transferred to lower concentrations used in biological assays.

Fluconazole content in samples studied in biological assays covered a wide range of concentrations starting from slightly below the concentration in the commercially available products in solution form (i.e. 2 mg/ml) reaching the maximum solubility of fluconazole in aqueous solutions[1] constituting the control solutions for biological assays.

Verapamil concentration was adjusted according to the literature regarding the study of efflux pumps.[2,3,4]

Table S1. Primers and fluorescent probes used in real-time RT-PCR.[5,6]

Gene (GenBank accession no.)	Primer or probe	Sequence (5' – 3')	Gene location
CgCDR1 (AF109723)	CDR1a	TAGCACATCAACTACACGAACGT	4500–4522
	CDR1b	AGAGTGAACATTAAGGATGCCATG	4647–4670
	CDR1pr	6FAM-TGCTGCTGCTTCTGCCACCTGGTT-TAMRA	4621–4644
CgCDR2 (AF251023)	CDR2a	GTGCTTTATGAAGGCTACCAGATT	164–187
	CDR2b	TCTTAGGACAGAAGTAACCCATCT	251–274
	CDR2pr	6FAM-TACCTTTGCGTGCTGGGCGTCACC-TAMRA	217–240
ERG11 (L40389)	ERGa	ATTGGTGTCTTGATGGGTGGTC	928–949
	ERGb	TCTTCTTGGACATCTGGTCTTTCA	1019–1042
	ERGpr	6FAM-ACTTCCGCTGCTACCTCCGCTTGG-TAMRA	955–978
URA3 (L13661)	URAa	GAAAACCAATCTTTGTGCTTCTCT	168–191
	URAb	CATGAGTCTTAAGCAAGCAAATGT	268–291
	URApr	6FAM-ACGTCACCACCACCAGCGAATTGT-TAMRA	194–217

6-FAM, 6-carboxyfluorescein; TAMRA, tetramethylrhodamine

2. Rheological studies

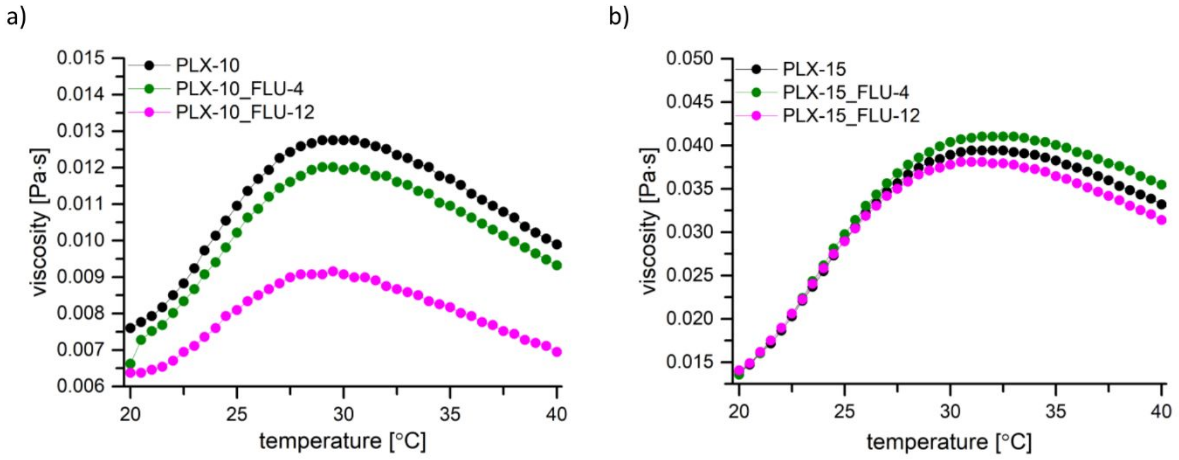

Figure S1. The effect of temperature on the viscosity in series PLX-10 (a) and PLX-15 (b), shear rates were 300 s^{-1} and 150 s^{-1}, respectively.

Table S2. The summary of the state of Pluronic® F-127 formulations at room temperature and under experimental conditions at particular polymer concentrations.

Pluronic® F-127 concentration	State of the formulation at the room temperature (15-25 °C)	State of the formulation at the experimental conditions (35 °C)
0.08 % w/v (PLX-0.08)	liquid	liquid
5 % w/v (PLX-5)	liquid	liquid
10 % w/v (PLX-10)	liquid	liquid
15 % w/v (PLX-15)	liquid	liquid
20 % w/v (PLX-20)	liquid	gel
25 % w/v (PLX-25)	gel	gel

3. Drug content in micellar solutions of Pluronic® F-127

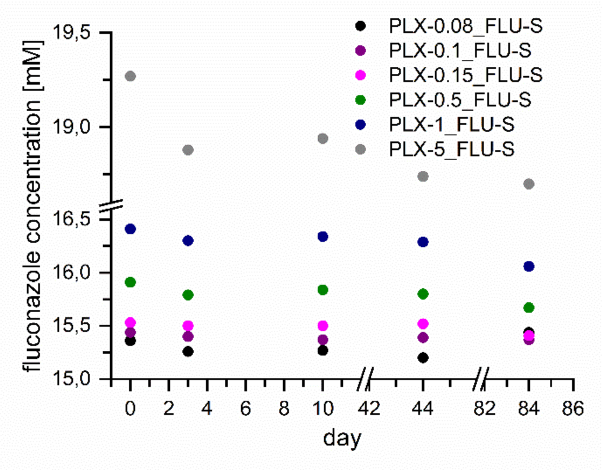

Figure S2. Fluconazole concentration in Pluronic® F-127 micellar solutions studied over 84 days.

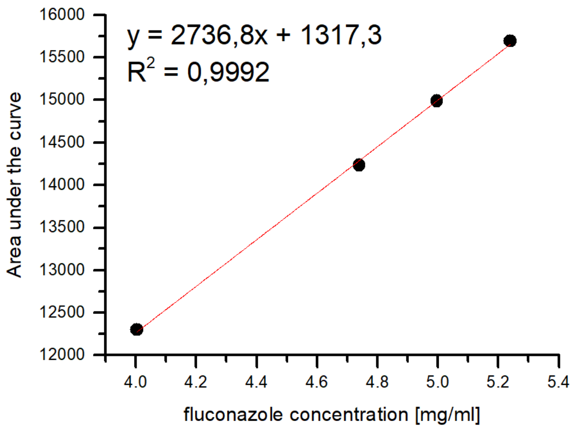

Figure S3. Calibration curve of fluconazole within 5% Pluronic® F-127.

Table S3. Area under the curve (above) and fluconazole concentration calculated based on the calibration curve (below) obtained by HPLC experiments.

Day	Area under the curve					
	PLX-0.08_FLU-S	PLX-0.1_FLU-S	PLX-0.15_FLU-S	PLX-0.5_FLU-S	PLX-1_FLU-S	PLX-5_FLU-S
0	14196	14255	14331	14650	15076	17471
3	14105	14227	14305	14550	14977	17141
10	14115	14203	14309	14597	15009	17195
44	14060	14220	14324	14559	14972	17029
84	14257	14199	14234	14451	14783	16991
Day	Fluconazole concentration [mg/ml]/[mM]					
	PLX-0.08_FLU-S	PLX-0.1_FLU-S	PLX-0.15_FLU-S	PLX-0.5_FLU-S	PLX-1_FLU-S	PLX-5_FLU-S
0	4,71/15,36	4,73/15,44	4,75/15,53	4,87/15,91	5,03/16,41	5,90/19,27
3	4,67/15,26	4,72/15,40	4,75/15,50	4,84/15,79	4,99/16,30	5,78/18,88
10	4,68/15,27	4,71/15,37	4,75/15,50	4,85/15,84	5,00/16,34	5,80/18,94
44	4,66/15,20	4,71/15,39	4,75/15,52	4,84/15,80	4,99/16,29	5,74/18,74
84	4,73/15,44	4,71/15,37	4,72/15,41	4,80/15,67	4,92/16,06	5,73/18,70

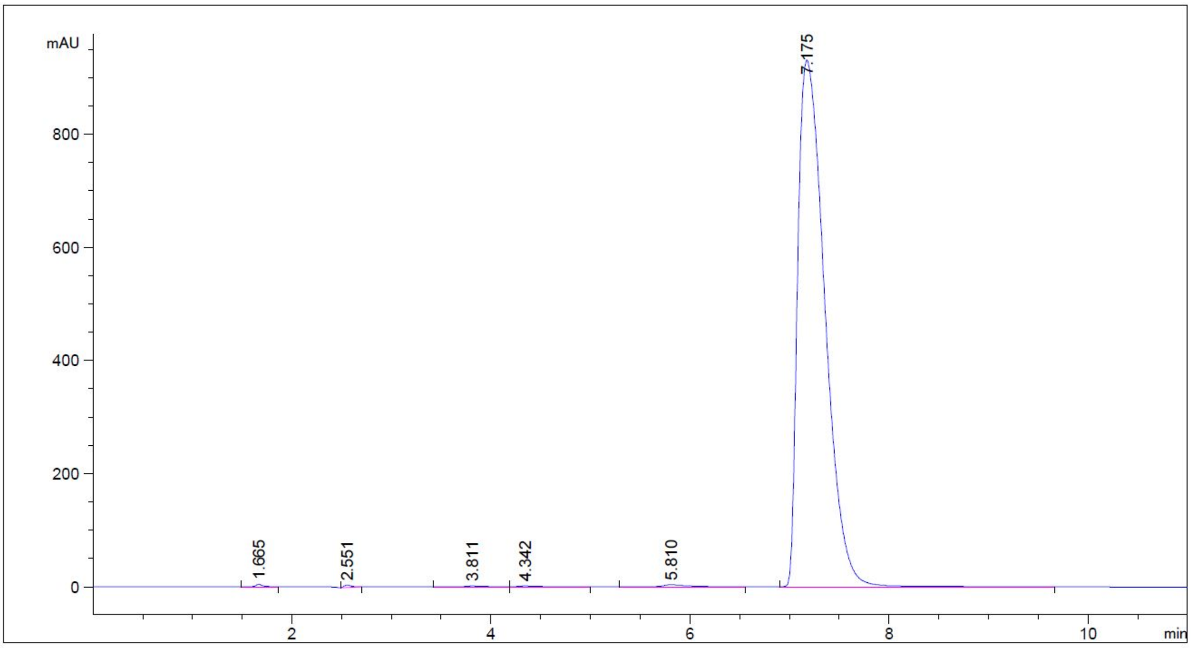

Figure S4. An example chromatogram of PLX-0.08_FLU-S (freshly prepared at day 0). Fluconazole peak visible at the retention time 7.175 minutes.

4. The statistical analysis of the results of broth microdilution method performed on resistant *Candida glabrata* strains

Table S4. One-dimensional significance tests for absorbance (series FLU-MIC, F127-0.08_FLU-MIC, F127-5_FLU-MIC, VER_FLU-MIC applied to resistant Candida glabrata), parameterization with sigma-constraints, decomposition of effective hypotheses.

Effect	Sum of Squares (SS)	Number of degrees of freedom	Mean Squares (MS)	F	p
Constant Term	345.8584	1	345.8584	54504.51	p<0.000001
Formulation	0.9932	3	0.3311	52.17	p<0.000001
Fluconazole	50.1419	5	10.0284	1580.39	p<0.000001
Strain	4.6065	14	0.3290	51.85	p<0.000001
Formulation* Fluconazole	1.0245	15	0.0683	10.76	p<0.000001
Formulation*Strain	0.8013	42	0.0191	3.01	p<0.000001
Fluconazole*Strain	3.6876	70	0.0527	8.30	p<0.000001
Formulation* Fluconazole*Strain	1.2763	210	0.0061	0.96	0.645170
Error	5.4127	853	0.0063		

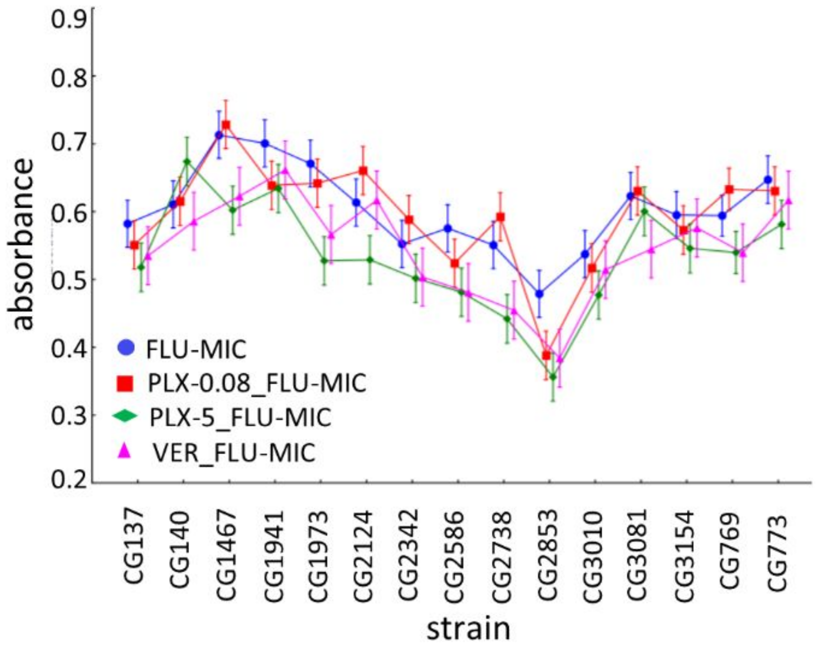

Figure S5. Results of broth microdilution method applied to resistant Candida glabrata derived from the Multivariate Analysis of Variance (MANOVA). Current effect: F(42, 853)=3.0067, p<0.00001, mean value ±95 % CI.

5. The statistical analysis of the results of broth microdilution method performed on all examined resistant *Candida* strains (*C. krusei, C. albicans, C. glabrata, C. tropicalis*)

The statistical analysis of the results performed on all the examined resistant *Candida* strains *(C. krusei, C. albicans, C. glabrata, C. tropicalis)* showed that there was no statistically significant difference in absorbance value upon the addition of polymer-based formulation at the lowest investigated Pluronic® F-127 concentration i.e. slightly above CMC loaded with fluconazole (Figure S5, FLU-MIC vs. F127-0.08_FLU-MIC, p = 0.138034). Increasing the concentration of Pluronic® F-127 in the sample of fluconazole up to 5.0 % w/v resulted in statistically significant decrease in absorbance followed by inhibited growth of yeasts (Figure S5, FLU-MIC vs. F127-5_FLU-MIC, p <0.000001). Considering respective strains, *C. albicans* ATCC MYA-574 displayed the largest measured difference in absorbance value between these two series (ESI, Figure S6 and Table S3, FLU-MIC vs. F127-5_FLU-MIC, p <0.000001), accompanied by *C. albicans* 1444 (ESI, Figure S4, p <0.000001), *C. glabrata* 2586, 2738, 2853, 1973, 1467, and 2124 (ESI, Figure S6, p = 0.000036, p <0.000001, p <0.000001, p = 0.000001, p = 0.000010, p = 0.000011, respectively).

Addition of verapamil (series VER_FLU-MIC) induced statistically significant decrease in growth of resistant fungi (Figure S5, FLU-MIC vs. VER_FLU-MIC, p = 0.011538). Taking into account particular strains statistically significant decrease in absorbance values after incubation yeasts with verapamil-fluconazole formulations comparing to FLU-MIC series was observed for the following strains: *C. glabrata* 2586 (ESI, Figure S6, p = 0.013918), 2738 (ESI, Figure S6, p = 0.007647), 2853 (ESI, Figure S6, p = 0.000196), 1973 (ESI, Figure S6, p = 0.004149), and 1467 (ESI, Figure S6, p = 0.010058).

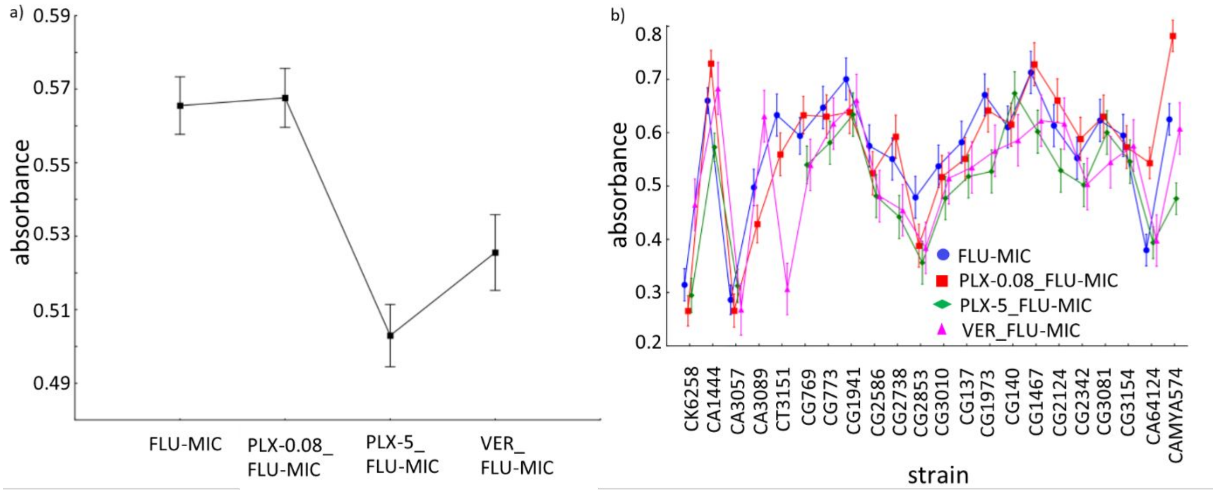

Figure S6. a) Results of broth microdilution method applied to resistant Candida strains (C. krusei, C. albicans, C. glabrata, C. tropicalis) derived from the Multivariate Analysis of Variance (MANOVA). Current effect: F(2, 1520) = 23.511, p <0.00001, mean value ±95 % CI. b) Results of broth microdilution method applied to resistant Candida strains (C. krusei, C. albicans, C. glabrata, C. tropicalis) derived from the Multivariate Analysis of Variance (MANOVA). Current effect: F(61, 1520) = 9.2569, p <0.00001, mean value ±95 % CI.

Table S5. One-dimensional significance tests for absorbance (series FLU-MIC, F127-0.08_FLU-MIC, F127-5_FLU-MIC, VER_FLU-MIC applied to resistant Candida strains: C. krusei, C. albicans, C. glabrata, C. tropicalis), parameterization with sigma-constraints, decomposition of effective hypotheses.

Effect	Sum of Squares (SS)	Number of degrees of freedom	Mean Squares (MS)	F	p
Constant Term		0			
Formulation	0.38515	2	0.192575	23.5113	p<0.000001
Fluconazole		0			
Strain	19.94150	19	1.049553	128.1384	p<0.000001
Formulation* Fluconazole	0.25766	10	0.025766	3.1457	0.000540
Formulation*Strain	4.62509	61	0.075821	9.2569	p<0.000001
Fluconazole*Strain	12.17893	95	0.128199	15.6517	p<0.000001
Formulation* Fluconazole*Strain	3.77053	305	0.012362	1.5093	0.000001
Error	12.44998	1520	0.008191		

6. The statistical analysis of the results of broth microdilution method performed on susceptible *Candida glabrata* strains

Table S6. One-dimensional significance tests for absorbance (series FLU-MIC, F127-0.08_FLU-MIC, F127-5_FLU-MIC, VER_FLU-MIC for susceptible Candida glabrata), parameterization with sigma-constraints, decomposition of effective hypotheses.

Effect	Sum of Squares (SS)	Number of degrees of freedom	Mean Squares (MS)	F	p
Constant Term	223.2629	1	223.2629	8076.942	p<0.000001
Formulation	0.3380	3	0.1127	4.075	0.006931
Fluconazole	0.5636	6	0.0939	3.398	0.002581
Strain	12.6711	7	1.8102	65.486	p<0.000001
Formulation* Fluconazole	1.1773	18	0.0654	2.366	0.001153
Formulation*Strain	5.1869	21	0.2470	8.935	p<0.000001
Fluconazole*Strain	4.5851	42	0.1092	3.949	p<0.000001
Formulation* Fluconazole*Strain	11.2870	126	0.0896	3.241	p<0.000001
Error	20.8974	756	0.0276		

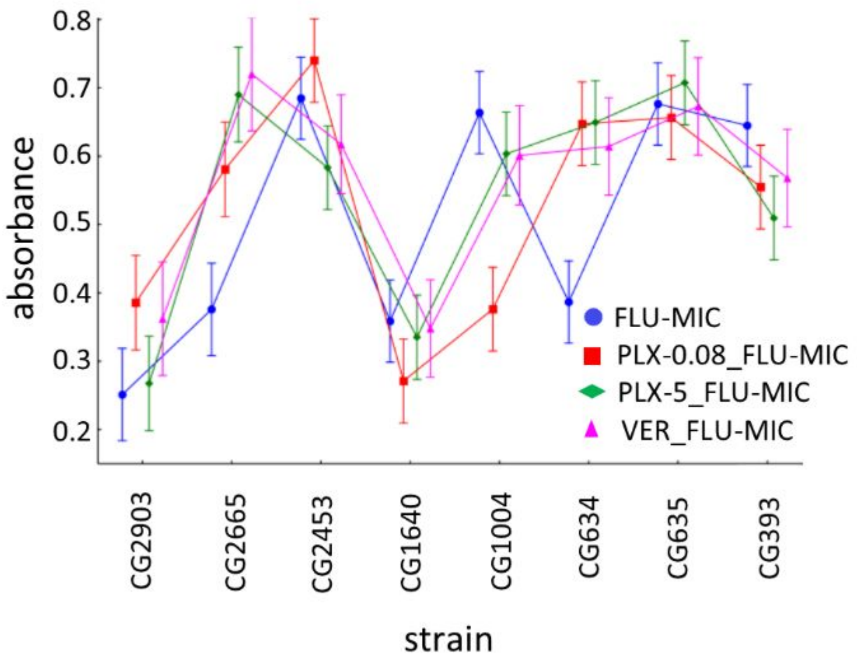

Figure S7. Results of broth microdilution method applied to susceptible Candida glabrata derived from the Multivariate Analysis of Variance (MANOVA). Current effect: $F(21, 756) = 8.9355$, $p < 0.00001$, mean value ±95 % CI.

7. Cup plate method results

Table S7. Antifungal activity of fluconazole and fluconazole loaded into 25 % w/v Pluronic® F-127 evaluated by cup plate method applied to resistant to fluconazole Candida strains (C. krusei, C. albicans and C. glabrata) (values presented with standard deviation, if no inhibition zone observed, the diameter of the well indicated in the table).

Strain	Inhibition zone [mm]			
	PLX-25_FLU-4	PLX -25_FLU-8	PLX -25_FLU-12	PLX -25_FLU-15
C. krusei ATCC 6258	7.0±0.0	13.5 ±1.0	16.9 ±1.7	18.6 ±1.0
C. albicans 3057	7.0±0.0	19.7 ±0.6	19.5 ±0.7	22.3 ±0.6
C. glabrata 2586	7.0±0.0	7.0±0.0	14.3 ±1.2	7.0±0.0
C. glabrata 2738	7.0±0.0	7.0±0.0	15.8 ±0.8	7.0±0.0
C. glabrata 2853	7.0±0.0	7.0±0.0	18.8 ±1.9	18.7 ±0.8
C. glabrata 3010	7.0±0.0	7.0±0.0	7.0±0.0	16.3 ±0.6
C. glabrata 1467	7.0±0.0	7.0±0.0	7.0±0.0	7.0±0.0
C. glabrata 2124	7.0±0.0	7.0±0.0	7.0±0.0	18.2 ±0.3
C. glabrata 137	not evaluated			
C. glabrata 140	not evaluated			

8. The statistical analysis of the results of cup plate method performed on resistant *Candida glabrata* strains

Table S8. One-dimensional significance tests for inhibition zone (series FLU, F127-10_FLU, F127-15_FLU, F127-20_FLU, resistant Candida glabrata), parameterization with sigma-constraints, decomposition of effective hypotheses.

Effect	Sum of Squares (SS)	Number of degrees of freedom	Mean Squares (MS)	F	p
Constant Term	95402.54	1	95402.54	54883.01	p<0.000001
Formulation	2474.34	3	824.78	474.48	p<0.000001
Fluconazole	49682.63	3	16560.88	9527.11	p<0.000001
Strain	14412.90	7	2058.99	1184.49	p<0.000001
Formulation* Fluconazole	512.47	9	56.94	32.76	p<0.000001
Formulation*Strain	4866.13	21	231.72	133.30	p<0.000001
Fluconazole*Strain	5193.26	21	247.30	142.27	p<0.000001
Formulation* Fluconazole*Strain	3031.70	63	48.12	27.68	p<0.000001
Error	1366,30	786	1,74		

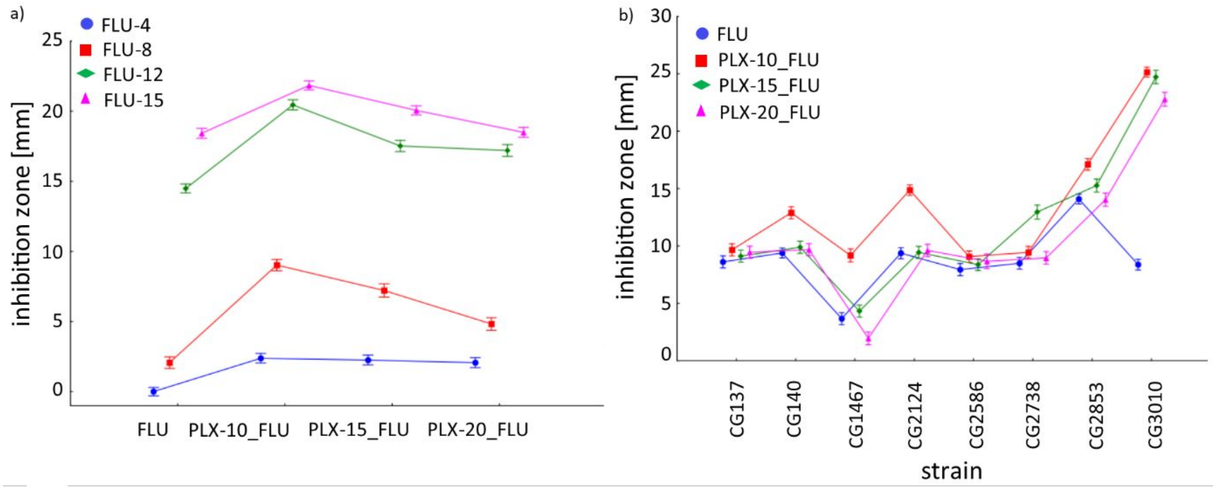

Figure S8. a) Results of cup plate method applied to resistant Candida glabrata derived from the Multivariate Analysis of Variance (MANOVA). Current effect: F(9,786) = 32.757, p <0.00001, mean value ±95 % CI. b) Results of cup plate method applied to resistant Candida glabrata derived from the

Multivariate Analysis of Variance (MANOVA). Current effect: F(21, 786) = 133.30, p <0.00001, mean value ±95 % CI.

9. The statistical analysis of the results of cup plate method performed on all examined resistant *Candida* strains (*C. krusei, C. albicans, C. glabrata*)

Statistical analysis performed on all examined resistant *Candida* strains *(C. krusei, C. albicans, C. glabrata)* showed that the inhibition zone was statistically significant larger when fluconazole was accompanied by block polymer regardless the polymer concentration (Figure S12, FLU vs. F127-10_FLU, p <0.000001, FLU vs. F127-15_FLU, p <0.000001, and FLU vs. F127-20_FLU, p <0.000001). The same phenomenon was observed when individual fluconazole concentration loaded to polymer samples was considered (ESI, Figure S12 and Table S8).

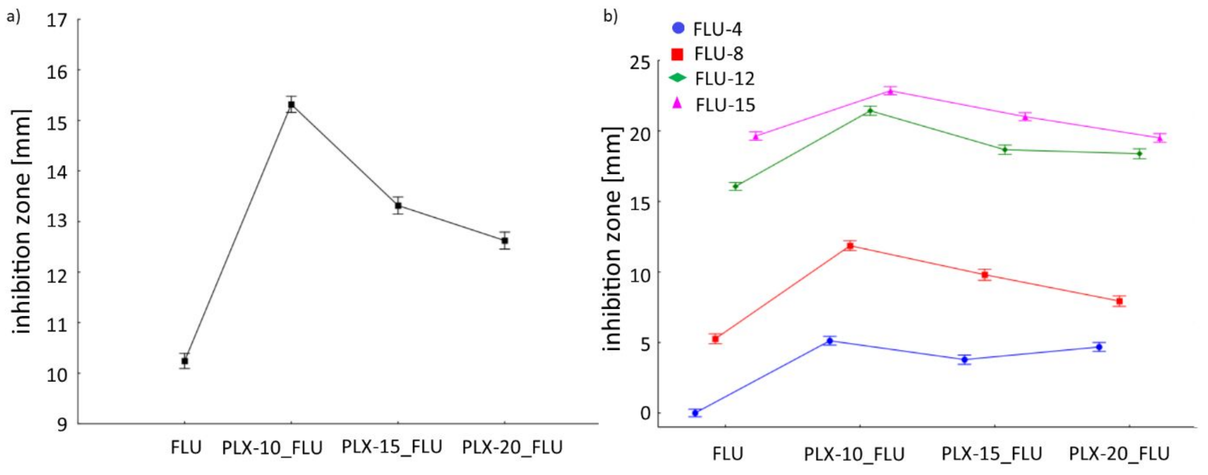

Figure S9. a) Results of cup plate method applied to resistant Candida strains (C. krusei, C. albicans and C. glabrata) derived from the Multivariate Analysis of Variance (MANOVA). Current effect: F(3, 1001) = 709,20, p <0.00001, mean value ±95 % CI. b) Results of cup plate method applied to resistant Candida strains (C. krusei, C. albicans, C. glabrata) derived from the Multivariate Analysis of Variance (MANOVA). Current effect: F(9, 1001) = 39.355, p <0.00001, mean value ±95 % CI.

Table S9. One-dimensional significance tests for inhibition zone (series FLU, F127-10_FLU, F127-15_FLU, F127-20_FLU, resistant Candida strains: C. krusei, C. albicans, C. glabrata), parameterization with sigma-constraints, decomposition of effective hypotheses.

Effect	Sum of Squares (SS)	Number of degrees of freedom	Mean Squares (MS)	F	p
Constant Term	164790.7	1	164790.7	97786.82	p<0.000001
Formulation	3585.5	3	1195.2	709.20	p<0.000001
Fluconazole	55050.6	3	18350.2	10889.02	p<0.000001
Strain	29703.1	9	3300.3	1958.42	p<0.000001

Formulation* Fluconazole	596.6	9	66.3	39.33	p<0.000001
Formulation*Strain	5325.7	27	197.2	117.05	p<0.000001
Fluconazole*Strain	7930.1	27	293.7	174.29	p<0.000001
Formulation* Fluconazole*Strain	5018.0	81	62.0	36.76	p<0.000001
Error	1686.9	1001	1.7		

10. Kinetics of antifungal activity

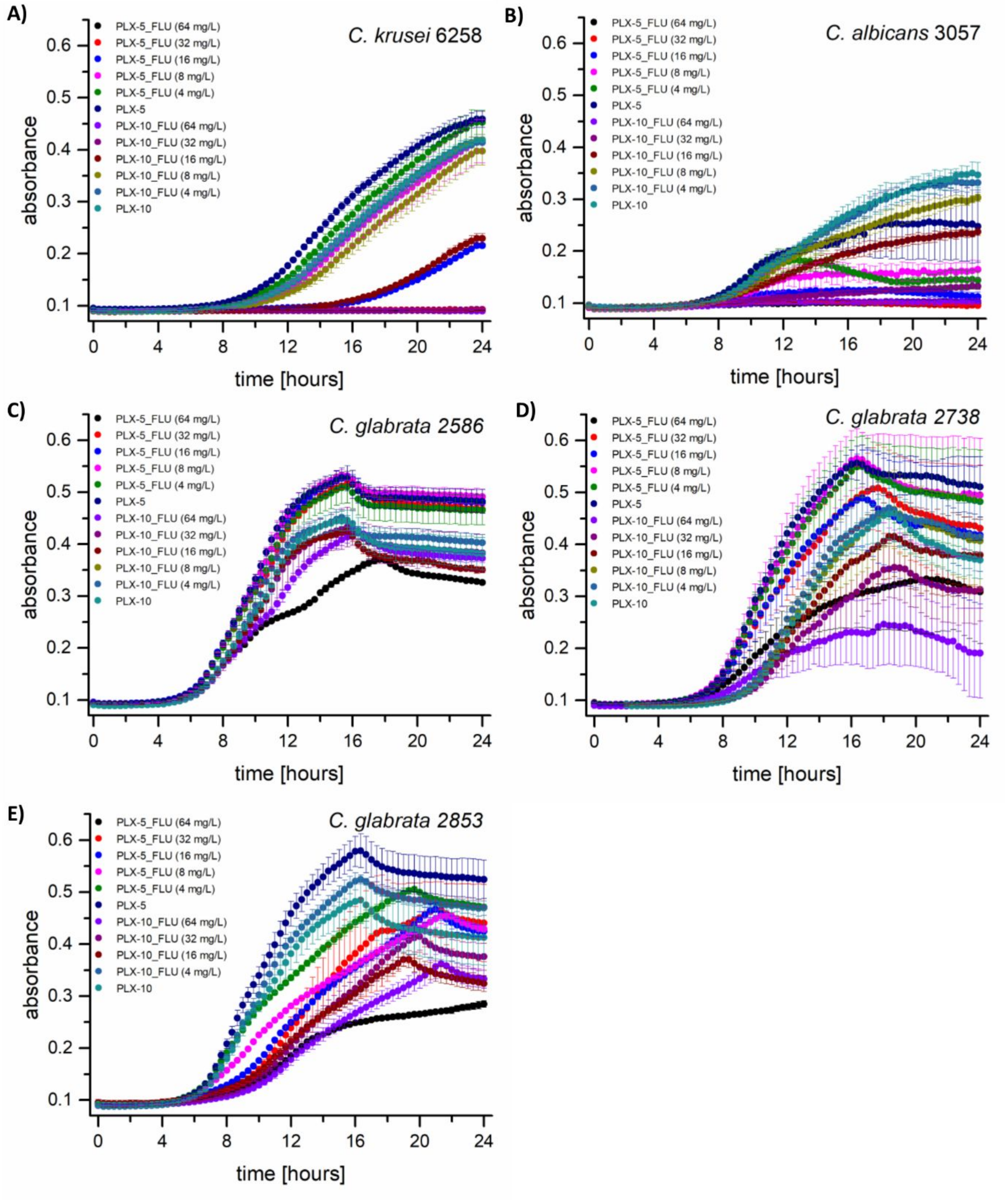

Figure S10. Growth curves of Candida over a 24 hours period.

11. Microscopic imaging

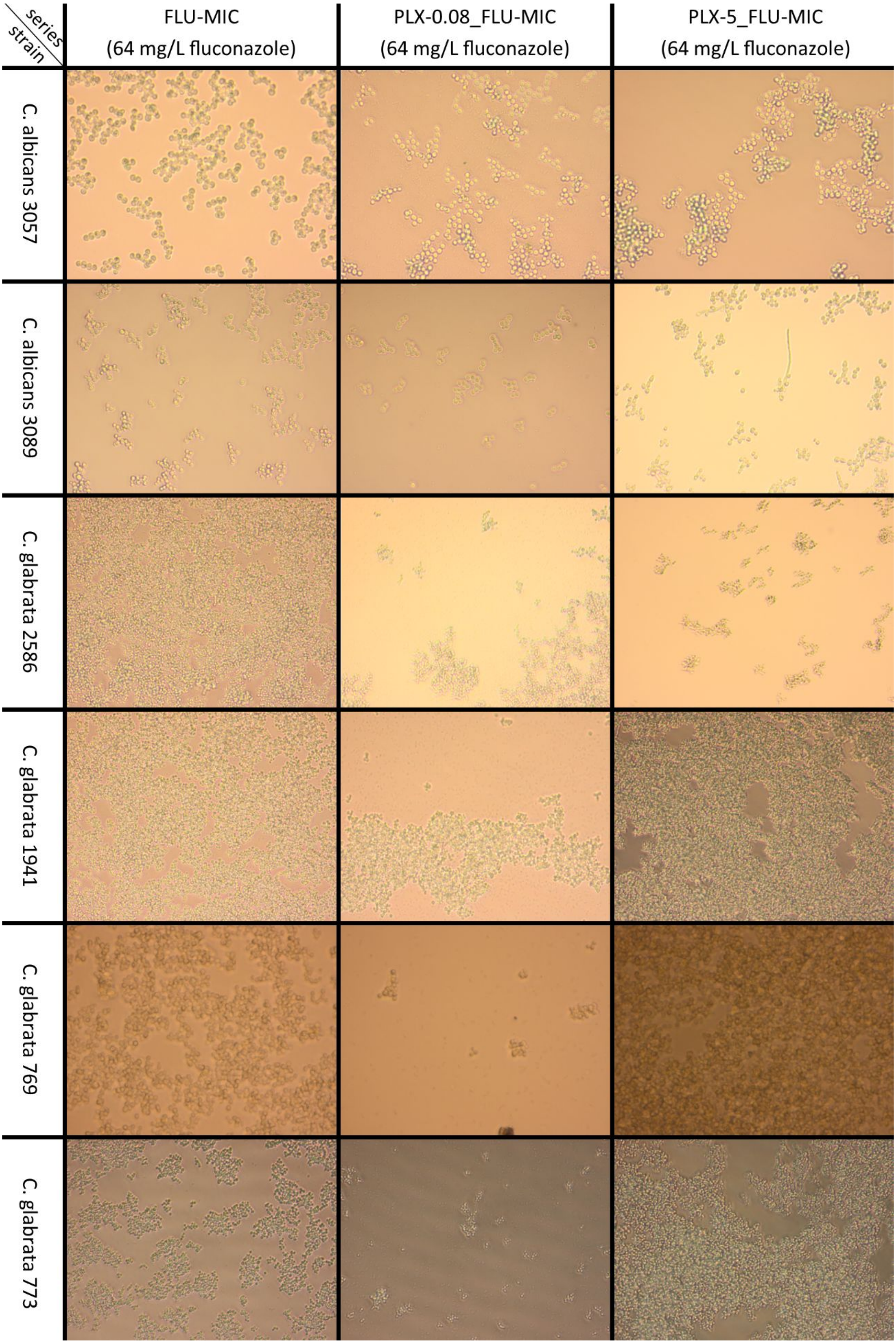

Figure S11. Microscopic images of Candida strains in series FLU-MIC, PLX-0.08_FLU-MIC and PLX-5_FLU-MIC (in wells of microtiter plate at fluconazole concentration 64 mg/L).

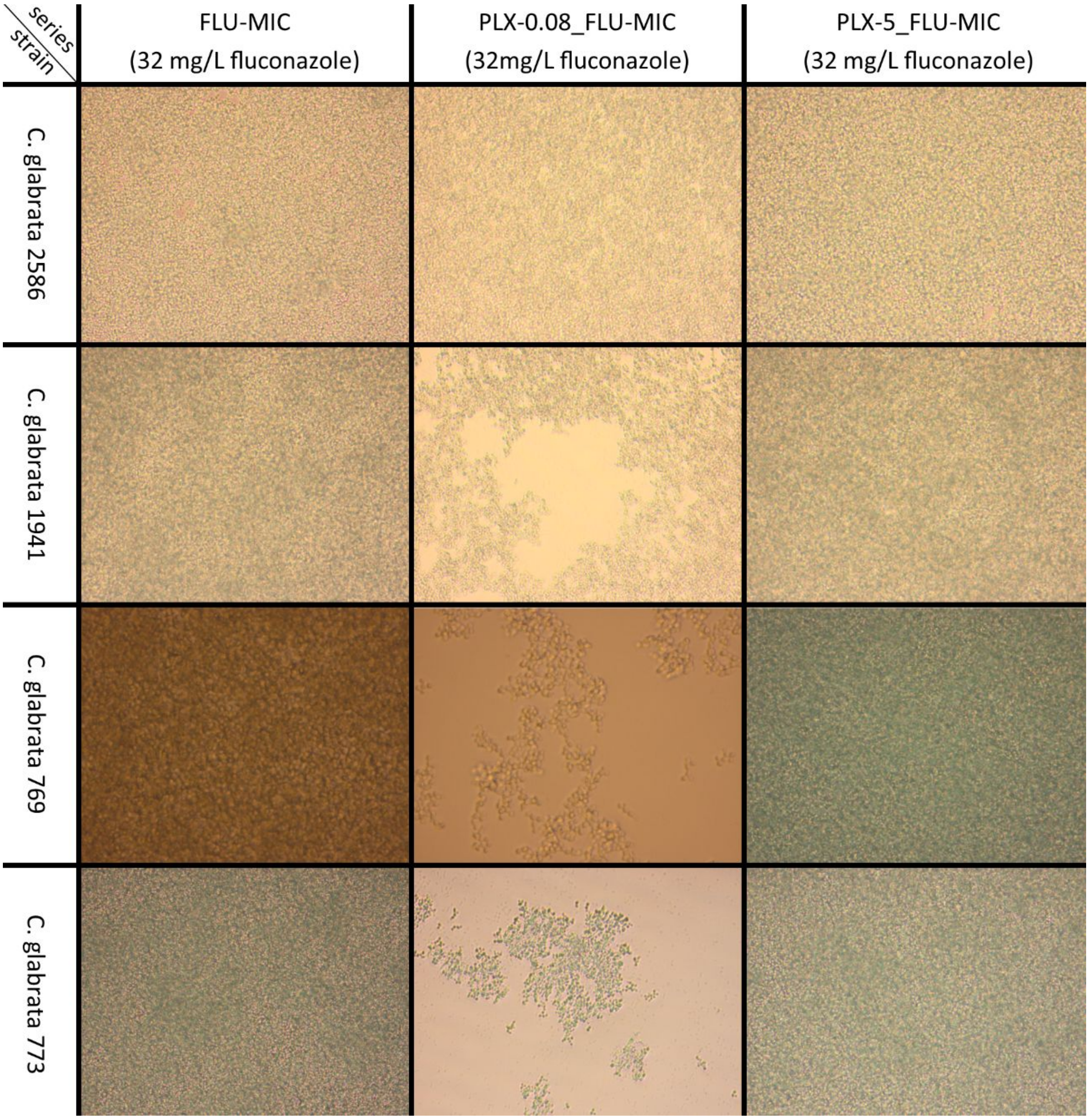

Figure S12. Microscopic images of Candida strains in series FLU-MIC, PLX-0.08_FLU-MIC and PLX-5_FLU-MIC (in wells of microtiter plate at fluconazole concentration 32 mg/L).

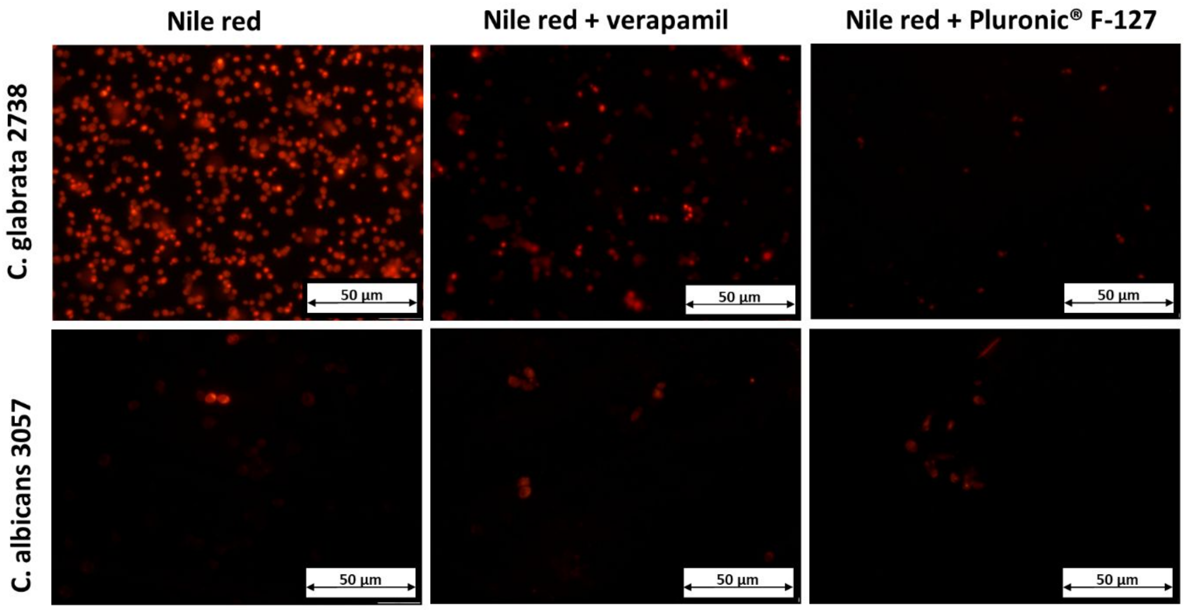

Figure S13. Candida strains stained with Nile red in the absence and presence of a reference substance (verapamil) and Pluronic® F-127.

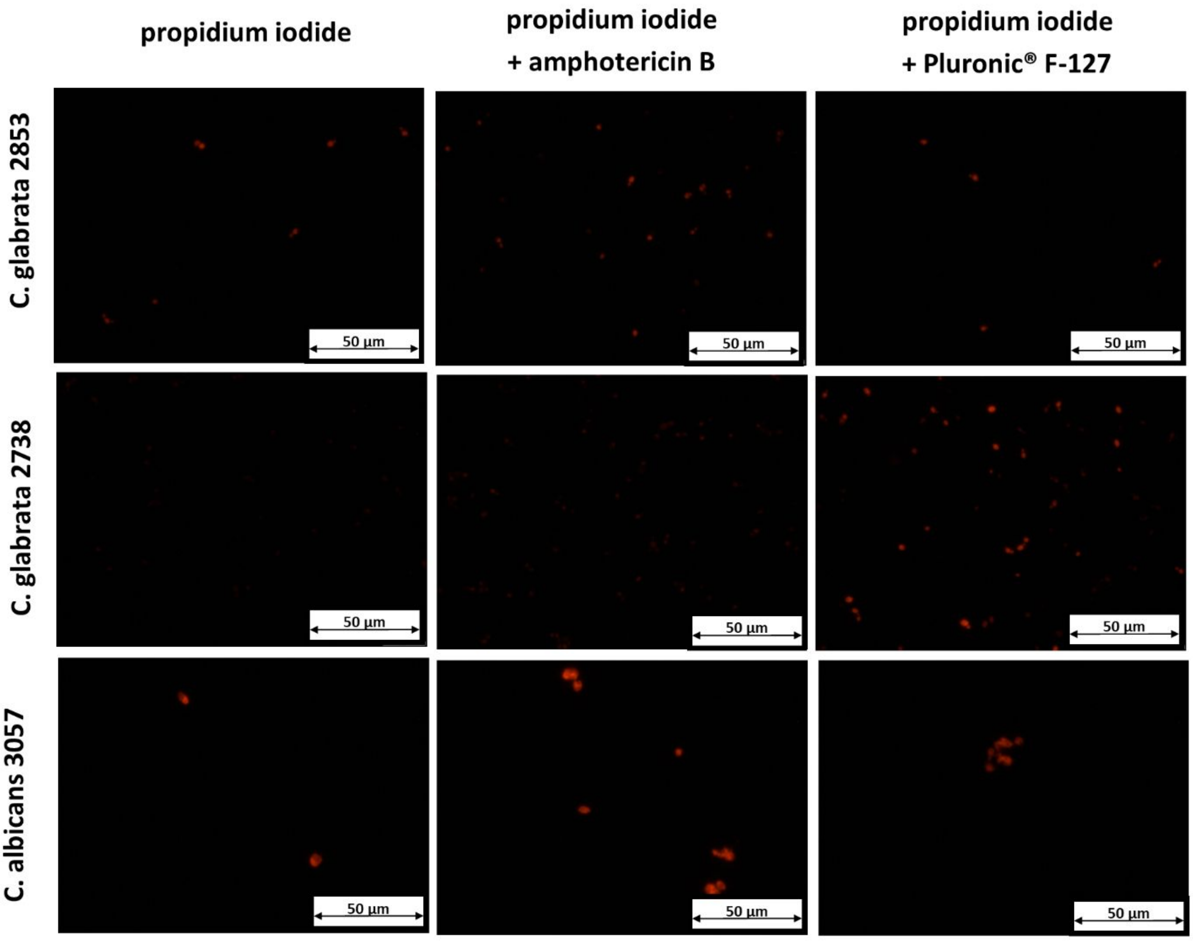

Figure S14. Candida strains stained with propidium iodide in the absence and presence of a reference substance (ampohotericin B) and Pluronic® F-127.

12. Gene expression analysis

Table S10. Expression level of the ERG11 gene in 15 C. glabrata resistant strains in comparison with the reference strain C. glabrata 1004 (characterized by MIC value for fluconazole of 8 mg/L).

Strain	ERG11 C_t (1)	ERG11 C_t (2)	ERG11 average C_t	URA3 C_t (1)	URA3 C_t (2)	URA3 average C_t	ΔC_t	$\Delta\Delta C_t$	$2^{-\Delta\Delta Ct}$
1004	25.71	26.19	25.95	27.85	26.76	27.31	-1.36	0.00	1.00
137	26.09	25.78	25.94	27.19	27.03	27.11	-1.18	0.18	0.88
2853	26.88	27.23	27.06	26.23	26.62	26.43	0.63	1.99	0.25
1467	26.09	26.73	26.41	27.83	27.05	27.44	-1.03	0.32	0.80
2342	26.31	25.83	26.07	26.06	25.72	25.89	0.18	1.54	0.35
769	26.72	26.79	26.76	26.35	26.39	26.37	0.38	1.74	0.30
3010	26.38	26.55	26.47	26.20	26.49	26.35	0.12	1.48	0.36
773	26.28	26.03	26.16	25.88	24.56	25.22	0.94	2.29	0.20
2738	26.82	27.07	26.95	25.92	26.57	26.25	0.70	2.06	0.24
140	26.44	26.38	26.41	27.82	28.46	28.14	-1.73	-0.38	1.30*
1941	26.43	26.18	26.31	27.91	28.18	28.05	-1.74	-0.39	1.31*
3154	27.45	27.39	27.42	28.32	27.85	28.09	-0.66	0.69	0.62
2124	26.97	26.34	26.66	28.39	27.58	27.99	-1.33	0.02	0.98
2586	27.43	27.38	27.41	27.34	26.87	27.11	0.30	1.66	0.32
1973	26.25	26.31	26.28	28.15	27.76	27.96	-1.68	-0.32	1.25*
3081	28.28	28.29	28.29	27.01	27.52	27.27	1.02	2.38	0.19

$\Delta C_t = C_{t(ERG11)} - C_{t(URA3)}$; $\Delta\Delta C_t = \Delta C_{t(investigated\ strain)} - \Delta C_{t(reference\ strain)}$; $2^{-\Delta\Delta Ct}$ indicates fold change in transcription of ERG11 gene normalized to URA3 in comparison to a reference strain C. glabrata 1004. Asterisk (*) indicates the range $1<2^{-\Delta\Delta Ct}<2$.

Table S11. Expression level of the CDR1 gene in 15 C. glabrata resistant strains in comparison with the reference strain C. glabrata 1004 (characterized by MIC value for fluconazole of 8 mg/L).

Strain	CDR1 C_t (1)	CDR1 C_t (2)	CDR1 average C_t	URA3 C_t (1)	URA3 C_t (2)	URA3 average C_t	ΔC_t	$\Delta\Delta C_t$	$2^{-\Delta\Delta Ct}$
1004	26.25	26.56	26.41	27.85	26.76	27.31	-0.90	0.00	1.00
137	25.05	25.69	25.37	27.19	27.03	27.11	-1.74	-0.84	1.79*
2853	26.35	27.28	26.82	26.23	26.62	26.43	0.39	1.29	0.41
1467	24.18	25.39	24.79	27.83	27.05	27.44	-2.66	-1.76	3.38**
2342	22.26	22.28	22.27	26.06	25.72	25.89	-3.62	-2.72	6.59**
769	25.79	25.19	25.49	26.35	26.39	26.37	-0.88	0.02	0.99
3010	23.38	24.2	23.79	26.20	26.49	26.35	-2.56	-1.66	3.15**
773	23.68	23.39	23.54	25.88	24.56	25.22	-1.69	-0.79	1.72*
2738	21.94	22.46	22.20	25.92	26.57	26.25	-4.05	-3.15	8.85**
140	26.66	26.05	26.36	27.82	28.46	28.14	-1.79	-0.89	1.85*
1941	26.05	26.23	26.14	27.91	28.18	28.05	-1.91	-1.01	2.01**
3154	27.05	26.71	26.88	28.32	27.85	28.09	-1.21	-0.31	1.24*
2124	27.65	27.96	27.81	28.39	27.58	27.99	-0.18	0.72	0.61
2586	26.18	25.74	25.96	27.34	26.87	27.11	-1.15	-0.25	1.19*
1973	25.71	24.65	25.18	28.15	27.76	27.96	-2.78	-1.88	3.67**
3081	25.52	25.29	25.41	27.01	27.52	27.27	-1.86	-0.96	1.95*

$\Delta C_t = C_{t(CDR1)} - C_{t(URA3)}$; $\Delta\Delta C_t = \Delta C_{t(investigated\ strain)} - \Delta C_{t(reference\ strain)}$; $2^{-\Delta\Delta Ct}$ indicates fold change in transcription of CDR1 gene normalized to URA3 in comparison to a reference strain C. glabrata 1004. Asterisk (*) indicates the range $1<2^{-\Delta\Delta Ct}<2$, whereas (**) indicates the values $2^{-\Delta\Delta Ct}>2$.

Table S12. Expression level of the CDR2 gene in 15 C. glabrata resistant strains in comparison with the reference strain C. glabrata 1004 (characterized by MIC value for fluconazole of 8 mg/L).

Strain	CDR2 C_t (1)	CDR2 C_t (2)	CDR2 average C_t	URA3 C_t (1)	URA3 C_t (2)	URA3 average C_t	ΔC_t	$\Delta\Delta C_t$	$2^{-\Delta\Delta Ct}$
1004	26.52	26.2	26.36	27.85	26.76	27.31	-0.95	0.00	1.00
137	25.55	25.17	25.36	27.19	27.03	27.11	-1.75	-0.81	1.75*
2853	25.39	25.22	25.31	26.23	26.62	26.43	-1.12	-0.18	1.13*
1467	25.3	24.66	24.98	27.83	27.05	27.44	-2.46	-1.52	2.86**
2342	24.62	24.07	24.35	26.06	25.72	25.89	-1.55	-0.60	1.52*
769	25.47	25.29	25.38	26.35	26.39	26.37	-0.99	-0.05	1.03*
3010	25.73	24.24	24.99	26.20	26.49	26.35	-1.36	-0.41	1.33*
773	24.28	23.91	24.10	25.88	24.56	25.22	-1.13	-0.18	1.13*
2738	22.6	22.38	22.49	25.92	26.57	26.25	-3.76	-2.81	7.01**
140	27.72	28.07	27.90	27.82	28.46	28.14	-0.25	0.70	0.62
1941	25.91	25.22	25.57	27.91	28.18	28.05	-2.48	-1.54	2.90**
3154	25.92	25.8	25.86	28.32	27.85	28.09	-2.23	-1.28	2.43**
2124	26.27	26.64	26.46	28.39	27.58	27.99	-1.53	-0.59	1.50*
2586	25.76	25.6	25.68	27.34	26.87	27.11	-1.43	-0.48	1.39*
1973	27.19	26.63	26.91	28.15	27.76	27.96	-1.05	-0.10	1.07*
3081	25.16	24.8	24.98	27.01	27.52	27.27	-2.29	-1.34	2.53**

$\Delta C_t = C_{t(CDR2)} - C_{t(URA3)}$; $\Delta\Delta C_t = \Delta C_{t(investigated\ strain)} - \Delta C_{t(reference\ strain)}$; $2^{-\Delta\Delta Ct}$ indicates fold change in transcription of CDR2 gene normalized to URA3 in comparison to a reference strain C. glabrata 1004. Asterisk (*) indicates the range $1<2^{-\Delta\Delta Ct}<2$, whereas (**) indicates the values $2^{-\Delta\Delta Ct}>2$.

13. References

(1) Malec, K.; Monaco, S.; Delso, I.; Nestorowicz, J.; Kozakiewicz-Latała, M.; Karolewicz, B.; Khimyak, Y. Z.; Angulo, J.; Nartowski, K. P. Unravelling the Mechanisms of Drugs Partitioning Phenomena in Micellar Systems via NMR Spectroscopy. *J. Colloid Interface Sci.* **2023**, *638*, 135–148. https://doi.org/10.1016/j.jcis.2023.01.063.

(2) Domingues Bianchin, M.; Borowicz, S. M.; da Rosa Monte Machado, G.; Pippi, B.; Stanisçuaski Guterres, S.; Raffin Pohlmann, A.; Meneghello Fuentefria, A.; Clemes Külkamp-Guerreiro, I. Lipid Core Nanoparticles as a Broad Strategy to Reverse Fluconazole Resistance in Multiple Candida Species. *Colloids Surfaces B Biointerfaces* **2019**, *175*, 523–529. https://doi.org/10.1016/j.colsurfb.2018.12.011.

(3) Pippi, B.; Lana, A. J. D.; Moraes, R. C.; Güez, C. M.; Machado, M.; de Oliveira, L. F. S.; Lino von Poser, G.; Fuentefria, A. M. In Vitro Evaluation of the Acquisition of Resistance, Antifungal Activity and Synergism of Brazilian Red Propolis with Antifungal Drugs on Candida Spp. *J. Appl. Microbiol.* **2015**, *118* (4), 839–850. https://doi.org/10.1111/jam.12746.

(4) Pinto E Silva, A. T.; Costa-De-Oliveira, S.; Silva-Dias, A.; Pina-Vaz, C.; Rodrigues, A. G. Dynamics of in Vitro Acquisition of Resistance by Candida Parapsilosis to Different Azoles. *FEMS Yeast Res.* **2009**, *9* (4), 626–633. https://doi.org/10.1111/j.1567-1364.2009.00508.x.

(5) Szweda, P.; Gucwa, K.; Romanowska, E.; Dzierżanowska-Fangrat, K.; Naumiuk, Ł.; Brillowska-Dąbrowska, A.; Wojciechowska-Koszko, I.; Milewski, S. Mechanisms of Azole Resistance among Clinical Isolates of Candida Glabrata in Poland. *J. Med. Microbiol.* **2015**, *64* (6), 610–619. https://doi.org/10.1099/jmm.0.000062.

(6) Sanguinetti, M.; Posteraro, B.; Fiori, B.; Ranno, S.; Torelli, R.; Fadda, G. Mechanisms of Azole Resistance in Clinical Isolates of Candida Glabrata Collected during a Hospital Survey of Antifungal Resistance. *Antimicrob. Agents Chemother.* **2005**, *49* (2), 668–679. https://doi.org/10.1128/AAC.49.2.668-679.2005.
